# Fluorescent Biofunctionalized Silver and Gold Nanoparticles: Bright Tools for Bioimaging Applications

**DOI:** 10.1021/acsomega.6c07048

**Published:** 2026-09-13

**Authors:** Elena Olivieri, Federica Bertelà, Sofia Lemaire, Miranda Parisi, Luca Battistini, Giovanna Borsellino, Daniela F. Angelini, Edyta Beyer, Barbara Wolanin, Tomasz Sobol, Valentin-Adrian Maraloiu, Giovanna Iucci, Chiara Battocchio, Iole Venditti

**Affiliations:** † Roma Tre University, Department of Sciences, via della Vasca Navale 79, 00146 Rome, Italy; ‡ Neuroimmunology Unit, Santa Lucia Foundation IRCCS, 00143 Rome, Italy; § National Institute of Materials Physics, 405A Atomistilor St., 077125 Bucharest, Romania; ∥ National Synchrotron Radiation Centre SOLARIS, Jagiellonian University, 30-392 Kraków, Poland

## Abstract

The aim of this work is to evaluate different nanostructured systems based on noble metal nanoparticles suitable for drug delivery and release related to the treatment of multiple sclerosis. To this end, four different systems are thoroughly characterized from a chemical-physical and structural perspective and, then, from a biological perspective. Gold and silver nanoparticles were synthesized and stabilized with different biocompatible ligands, including poly­(ethylene glycol), citrate, and cysteine, to modulate their colloidal stability and surface reactivity. UV–Vis spectroscopy revealed characteristic plasmonic absorption bands at about 400 nm for silver and nearly 550 nm for gold nanoparticles. Morphological analyses, performed by Transmission Electron Microscopy, and colloidal stability, studied by Dynamic Light Scattering, highlighted differences in size distribution and polydispersity among the systems. The nanoparticles were subsequently functionalized with fluorescein isothiocyanate, a fluorescent dye, to enable their detection and tracking within cells using an accessible analytical technique appropriate for the study of immune cells, such as flow cytometry. Nanoparticle surface properties were thoroughly investigated by means of Fourier-transform infrared, synchrotron radiation-induced X-ray photoelectron, and near edge X-ray absorption fine structure spectroscopies. Flow cytometry analyses demonstrated efficient nanoparticle–cell interaction with peripheral blood mononuclear cells, and a cell viability assay performed with the improved system highlighted the potential for applications in drug delivery and bioimaging.

## Graphical abstract

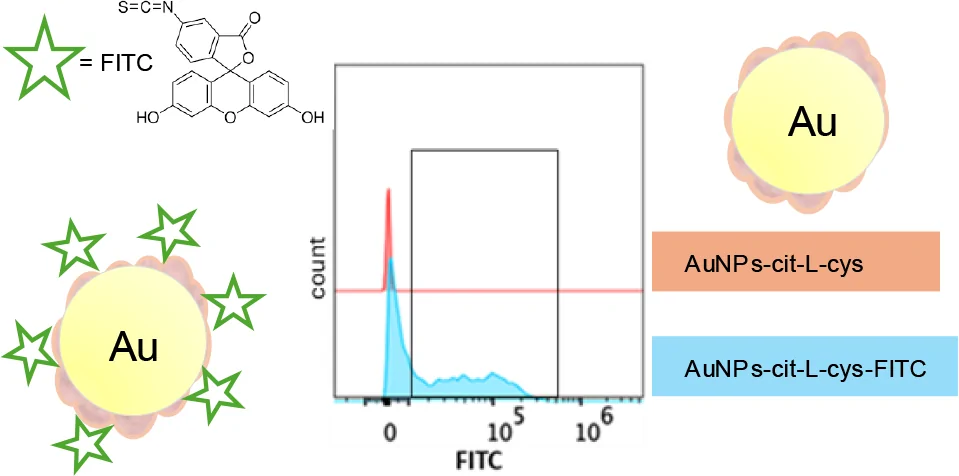

## 1 Introduction

Metal nanoparticles show very attractive chemical-physical properties that, in the past decade, have made them widely used for the development of advanced applications in various fields, from energy to catalysis, to optics, to biomedicine.^1–6^ Indeed, as drug delivery systems, metal nanoparticles demonstrate several advantages, especially when compared with polymeric nanoparticles. Polymeric nanoparticles excel at high-capacity encapsulation, controlled release, and optical properties when nanosized.^7–10^ Metal nanoparticles, on the other hand, exhibit superior thermal and chemical stabilities, unique optoelectronic properties, and greater resistance to enzymatic degradation. These particles do not oxidize, corrode, or readily degrade in biological fluids, ensuring that their internal structure remains intact during circulation. Furthermore, they withstand severe sterilization and storage conditions, whereas polymeric particles may suffer from polymer chain breakage or premature drug loss over time. Among others, gold and silver nanoparticles (AuNPs and AgNPs) have been particularly successful in the past decade.^11–15^ In fact, the possibility of their *ad hoc* synthesis allows them to design and optimize their preparation based on the various needs, also considering the sustainability of the synthetic approach.^16–20^ AuNPs and AgNPs have different characteristics regarding shape, size, and surface functionalization that closely influence the chemical and physical characteristics, including the optical properties.^21,22^

Thanks to the phenomenon of localized surface plasmon resonance (LSPR), the AuNPs and AgNPs can be easily studied and monitored; LSPR originates from the oscillation of free electrons in the conduction band of a metal.^23^ The light corresponding to the oscillation frequency is absorbed by the metal surface, resulting in a local electromagnetic field increase. Since nanoparticles (NPs) are smaller than the wavelength of the incident light, the charge density is redistributed and oscillates locally around the NP. This property is widely used in sensors, optical devices, lenses, antennas, data storage, waveguides, microscopy, and many other fields.^24^

For AuNPs and AgNPs, their versatility in binding different molecules on the surface is surprising, both to stabilize the NPs themselves or increase their biocompatibility, but above all, in binding drugs on the surface and becoming efficient, effective, and controlled drug delivery systems (DDSs).^25^ In fact, AuNPs and AgNPs are widely used as DDSs to deliver drugs to specific sites and make them more bioavailable, thereby reducing systemic side effects.

Among the two types of noble metal nanoparticles, gold nanoparticles are more extensively used in biomedicine due to their higher colloidal and chemical stabilities, which lead to reduced reactivity and consequently greater biocompatibility. In addition, gold offers greater potential for surface functionalization owing to its favorable chemical properties and strong affinity for thiol and amine groups, facilitating targeted and multifunctional applications.^26,27^

These characteristics are particularly relevant for numerous diseases for which there are still no effective therapies, often because of the poor bioavailability of drugs linked to their rapid dispersion and low chemical stability. As a result, only a limited fraction of the active ingredient reaches the therapeutic site, making it necessary to administer high doses with an increased risk of systemic side effects.

This aspect is particularly critical in the case of neurodegenerative diseases, where the therapeutic target is in the central nervous system (CNS), which is strongly protected by the blood-brain barrier (BBB). The BBB limits the passage of numerous drugs due to their chemical nature (ionized and nonlipophilic molecules) or their size, resulting in subtherapeutic concentrations in the CNS and, consequently, reduced therapeutic efficacy. In this context, nanoparticle-based drug delivery systems represent a highly innovative approach to overcoming the limitations of the traditional drug administration. Nanoparticles can improve drug stability, facilitate BBB crossing,^28^ and reduce systemic side effects thanks to more targeted release.^29^ Furthermore, the possibility of functionalizing nanoparticles with fluorescent dyes is an additional advantage, as it allows their biological behavior, cellular distribution, and interaction with tissues to be studied using techniques such as cytofluorimetry. Overall, nanoparticles offer an effective strategy for both improving the therapeutic efficacy and detailed analysis of the biological mechanisms involved.

In this work, gold and silver nanoparticles (AuNPs, AgNPs) functionalized with polyethylene glycol thiolate (PEG-SH) or citrate (cit) and l-cysteine (l-cys) were synthesized and thoroughly characterized in order to identify the best drug delivery system for the treatment of multiple sclerosis. Comparative structural and chemical-physical studies of these four systems, with and without fluorescein isothiocyanate (FITC) dye conjugated, were performed to evaluate how surface functionalization and nanoparticle composition influence their properties. The feasibility of monitoring nanoparticle behavior was assessed using flow cytometry with peripheral blood mononuclear cells (PBMCs). Moreover, for the best systems, cell viability was performed using PBMCs. Overall, this work lays the groundwork for future biomedical applications by combining nanoparticle engineering with advanced analytical techniques.

## 2 Experimental Section

### 2.1 Materials and methods

Sodium citrate (Na_3_C_6_H_5_O_7_, ≥ 99.0%, Sigma-Aldrich), l-Cysteine (C_3_H_7_NO_2_S, ≥ 97.0%, Sigma-Aldrich), tetrachloroauric­(III) acid trihydrate (HAuCl_4_·3H_2_O, ≥99.9%, Sigma-Aldrich), sodium borohydride (NaBH_4_, 99.99%, Sigma-Aldrich), silver nitrate (AgNO_3_, 99.9%, Carlo Erba), sodium hydroxide (NaOH, ≥ 97.0%, Sigma-Aldrich), Poly­(ethylene glycol), 2-mercaptoethyl ether acetic acid (PEG-S, HSC_2_H_4_O­(C_2_H_4_O)­nCH_2_CO_2_H, Sigma-Aldrich), fluorescein isothiocyanate (FITC, 90%, Sigma-Aldrich). Ficoll-Paque PLUS density gradient media (density max. 1.078 g/mL, cytiva), Growing medium (RPMI-1640 with l-glutamine and sodium bicarbonate, liquid, sterile-filtered, suitable for cell culture, Sigma-Aldrich), Human Serum (Sigma-Aldrich), Brillant Stain Buffer (BD Biosciences), Dulbecco’s Phosphate Buffered Saline (DPBS, Sigma-Aldrich).

### 2.2 Synthesis of AuNPs and AgNPs Functionalized with Citrate and l-Cysteine

The AuNPs stabilized with citrate and l-cysteine (AuNPs-cit-l-cys) have been prepared and characterized in analogy to literature reports.^9,30,31^ Briefly: 25 mL of l-cysteine water solution (0.002 M), 10 mL of citrate water solution (0.01 M), and 2.5 mL of tetrachloroauric acid water solution (0.05 M) were added sequentially in a 100 mL flask, provided with a magnetic stir. The solution was degassed with Ar for 10 min, and then 4 mL of sodium borohydride solution (0.05 mol) was added. After 2 h, the brown solid was recollected and purified by centrifugation (13,000 rpm, 10 min, 4 times with deionized water).

### 2.3 Synthesis of AuNPs and AgNPs Functionalized with PEG

The same concentration of gold and silver was used for both syntheses of AuNPs-PEG and AgNPs-PEG nanoparticles, in analogy with the literature report.^32^ Briefly: water solution of HAuCl_4_ or AgNO_3_ (0.025 M, 1.25 mL) was added to 12.5 mL of PEG-SH and stirred at room temperature for 10 min after the addition of 20 mL of NaOH (0.008M). An aqueous solution of NaBH_4_ (0.01 M, 6 mL) was added to the mixture. The as-prepared NP suspension was centrifuged at 13000 rpm for 20 min (three times).

### 2.4 NPs Labeled with FITC

All types of nanoparticles, synthesized as described above (Scheme 1.a), were functionalized with fluoresceine isothiocyanate (FITC) (Scheme 1.b). The functionalization protocol was: 2 mg of AuNPs (or AgNPs) were contacted with 5 mL of FITC (5 × 10^–5^ M water solution). After 24 h of stirring, the solution was centrifuged at 13000 rpm for 15 min. Supernatant and nanoparticles (pellet) were analyzed by UV–vis; the fluorescence signal of nanoparticles functionalized with FITC was also evaluated by fluorescence spectroscopy.

**Scheme 1:**
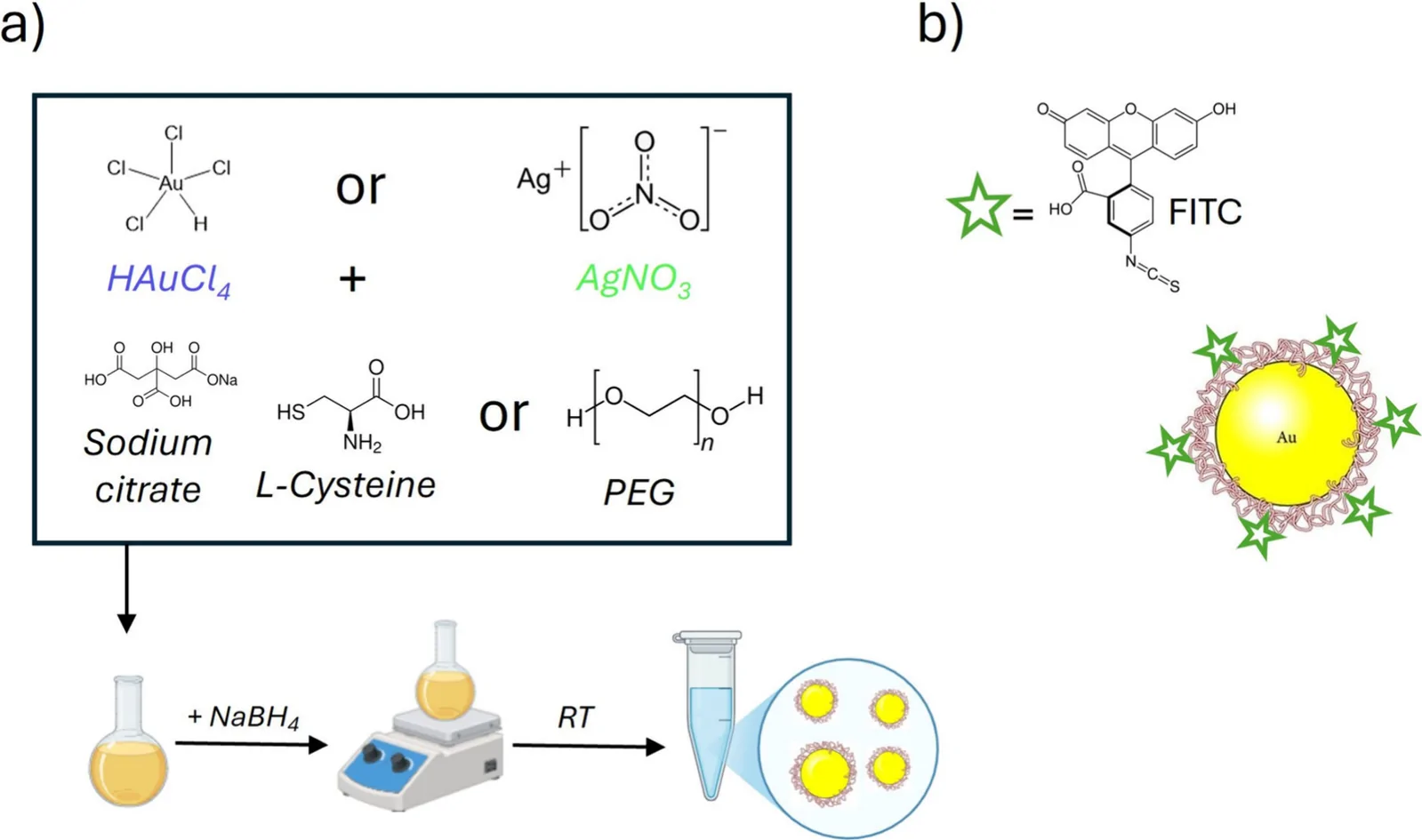
a) Synthesis Protocol Used for Au and AgNPs-cit-l-cys and Au and AgNPs-PEG; b) Interaction between FITC and the Nanoparticles

### 2.5 Biological Tests on Cell Viability

Primary Peripheral Blood Mononuclear Cells (PBMCs) were isolated from freshly collected whole blood by Ficoll–Paque density gradient centrifugation. Cells were seeded at a concentration of 5 × 10^5^ cells/well in U-bottom 96-well plates in RPMI medium supplemented with 5% human serum and penicillin–streptomycin. Cells were incubated at 37 °C with or without different concentrations (10, 25, and 50 μg/mL) of citrate–cysteine (Cit-Cys) and PEG nanoparticles (NPs), either FITC-functionalized or nonfunctionalized (used as an additional control), for 3, 18, and 24 h. At each time point, cells were harvested and transferred to V-bottom 96-well plates for staining with a viability dye (LIVE/DEAD Fixable Aqua Dead Cell Stain Kit, Invitrogen) for 20 min at room temperature (RT). Cells were subsequently fixed with formaldehyde and acquired at flow cytometer (Beckman Coulter).

### 2.6 Chemico-Physical Characterizations

*Ultraviolet–visible spectroscopy (UV–Vis)*: spectra were recorded with a Shimadzu 2401 PC UV–vis spectrophotometer and using quartz cuvettes with an optical path of 1 cm. All spectra were collected in the range 200–800 nm, with a resolution of 1 nm. Double-distilled water was used as the solvent.

*Fluorescence spectrophotometer*: spectra were recorded with an AGILENT TECHNOLOGY Cary with xenon flash lamp (80 Hz). For this type of analysis, the sample was measured inside quartz cuvettes with an optical path of 1 cm.

The size, size distribution, and colloidal stability of nanoparticles in aqueous solution were studied by *Dynamic Light Scattering* (DLS) using a Zetasizer Nanoseries Malvern Ultra RED instrument, at a temperature of 25.0 ± 0.2 °C. All measurements were performed at least in triplicate. Correlation data were acquired and adapted by analogy to our previous works.^33^

*Synchrotron Radiation-induced X-ray Photoelectron Spectroscopy (SR-XPS)* experiments were carried out on AuNPs-cit-l-cys, AuNPs-cit-l-cys-FITC, AuNPs-PEG, pristine PEG-SH, and AuNPs-PEG-FITC and at the SuperESCA beamline at the ELETTRA synchrotron facility of Trieste (Italy). XPS data were collected in fixed analyzer transmission mode (pass energy = 20 eV), with the monochromator entrance and exit slits optimized at 30 and 20 μm, respectively. For the C 1s, N 1s, and O 1s spectral regions, a photon energy (PE) of 600 eV was used; for the Au 4f and S 2p spectral regions the PE was 260 eV, as to maximize signal intensity. The energy resolution was ΔE = 0.25 eV for the entire energy range. Calibration of the energy scale was made referencing the spectra to the C 1s core level signal of aliphatic carbons, found at 285.00 eV, for signals acquired with photon energy = 600 eV; for signals acquired at photon energy = 260 eV spectra were calibrated using the Au 4f_7/2_ core level signal of metallic gold, found at 83.8 eV. Curve-fitting analysis of the C 1s, O 1s, N 1s, Au 4f, and S 2p spectra was done using Gaussian curves as fitting functions. The S 2p_3/2_,_1/2_ doublets were fitted using the same full width at half-maximum (fwhm) for both components, a spin–orbit splitting of 1.2, and a branching ratio (2p_3/2_/2p_1/2_) of 2. For the Au 4f_7/2_,_5/2_ doublets, a splitting of 3.7 eV, and a branch ratio of Au 4f_7/2_/Au 4f_5/2_ of 4/3 and the same fwhm values for both spin–orbit components were applied. When several species were identified in a spectrum, the same fwhm value was set for all individual photoemission bands.

SR-XPS measurements of AgNPs-cit-l-cys, AgNPs-cit-l-cys-FITC, AgNPs-PEG, and AgNPs-PEG-FITC were performed at the Near-Ambient Pressure XPS, NAPP branch of CIRCE beamline of the ALBA Synchrotron Light Source, Barcelona (Spain), an undulator beamline wherein a plane mirror and three diffraction gratings are used to cover the source energy range from 100 to 2000 eV;^34^ to easily compare the HR-XPS data collected at the two different beamlines, the measurement parameters were selected in analogy with the ones used at SuperESCA; i.e., PE = 260 and 600 eV, energy resolution of about ΔE = 0.1 eV. Curve-fitting analysis of the C 1s, N 1s, S 2p core-level spectra was carried out as described above for AuNPs sample; for Ag 3d_5/2_,_3/2_ doublets, a splitting of 6.0 eV, and a branch ratio of Ag 3d_5/2_/Ag 3d_3/2_ of 3/2 and the same fwhm values for both spin–orbit components were applied. When several species were identified in a spectrum, the same fwhm value was set for all individual photoemission bands.

*Near Edge X-ray Absorption Fine Structures (NEXAFS)* spectroscopy measurements at the C and N K edges were performed at the PHELIX beamline located at the National Synchrotron Radiation Centre SOLARIS in Krakow (Poland).^35^ PHELIX operates in the soft X-ray energy range of 40–2000 eV, and its source is an elliptically polarizing undulator.

Investigations were carried out in Total Electron Yield mode on thin films of AgNPs-cit-l-cys-FITC and AgNPs-PEG-FITC deposited by drop-casting from mother solution on gold-coated silicon substrates; pristine FITC was also analyzed for comparison. The angle between the sample surface and the impinging SR radiation was 20° (grazing incidence) in order to maximize signal intensity. Experimental spectra were normalized by dividing the sample spectrum by the spectrum of a clean gold surface; subsequently a straight line fitting the part of the spectrum below the edge was subtracted and the value at 320.00 and 430.00 eV for C and N, respectively, was assessed to 1. Spectra were energy-referenced to the π* resonance of aromatic rings located at 285.0 eV.^36–38^

*Fourier Transform Infrared (FTIR)* measurements were performed by means of a VECTOR 22 (Bruker) FT-IR interferometer operating in the wavenumber range 4000–400 cm^–1^, with a resolution of 1 cm^–1^, equipped with a DTGS detector. NPs samples were deposited as thin films by casting from aqueous solutions onto gold-coated silicon substrates. RAIRS (Reflection Absorption Infrared Spectroscopy) spectra of the NPs films were recorded by means of a Specac P/N 19650 series monolayer/grazing angle accessory at incidence angle of 70° with respect to the normal to the sample surface. The FTIR spectrum of FITC was recorded in transmittance mode from KBr pressed pellets.

For *transmission electron microscopy (TEM)* analysis, images were obtained either with a Philips EM 208S instrument (FEI-Thermo Fisher, Waltham, MA, USA), operating at 100 kV, equipped with a Mega-view II SIS Olympus camera or with a probe-corrected JEOL JEM ARM200F microscope (JEOL Ltd., Tokyo, Japan) operated at 200 kV, equipped with a Gatan (Gatan Ametek, Pleasanton, CA, USA) Ultrascan CCD camera. Samples were drop-cast on Formvar/carbon-supported copper grids, and after a few minutes, the excess liquid was blotted with filter paper, and then the samples were examined. *TEM* was used to analyze the microstructures of materials. TEM works by transmitting an electron beam through a thin sample and detecting the electrons passing through the sample. The electrons passing through the sample are projected onto a fluorescent screen or detector that produces an image. TEM operates under high-vacuum conditions and requires very thin samples. The resolution of TEM is determined by the wavelength of the electrons used in the beam. The wavelength of electrons is much shorter than that of visible light, allowing for much higher-resolution images. The typical TEM resolution is in the range of a few tenths of a nanometer to a few nanometers.^39^

*Flow cytometry* samples were acquired on a fully equipped CytoFLEX LX (Beckman Coulter). Quality control beads (Beckman Coulter) were used daily to check and standardize the instrument performance. Data were analyzed with FlowJo v.10.7.

## 3 Results and Discussion

### 3.1 Synthesis and Characterization of Functionalized AuNPs and AgNPs

The NPs used in this work are AuNPs or AgNPs stabilized during the synthesis with citrate and l-cysteine or with PEG-SH. In fact, PEG is a long, hydrophilic, and highly flexible polymer chain. When bound to the surface of a nanoparticle, it forms a bulky, hydrated, dynamic shield. This creates a steric barrier that physically prevents the nanoparticles from colliding and aggregating even in solutions with high salt concentrations or pH variations. Furthermore, using a thiolated PEG ensures a strong covalent bond to the surface of the metal particles.

On the other hand, citrate and l-cysteine contain multiple carboxyl groups (−COOH/-COO^–^). In neutral or basic aqueous solutions, these groups deprotonate, imparting a strong and uniform negative charge to the surface of the nanoparticle. This charge creates a repulsive electrostatic field, forcing neighboring nanoparticles to repel each other. Furthermore, cysteine has a dual functionality: it possesses a thiol group (-SH) that binds strongly to the surface of the nanoparticle. At the same time, its exposed amino (−NH_2_) and carboxyl (−COOH) groups remain available to interact with the environment, stabilize nearby biomolecules, or enable downstream chemical conjugation. For the synthesis of all types of these NPs, established experimental protocols described in scientific literature were adopted.^29–32^ The initial synthesis procedures based on well-established protocols served as a foundation from which the synthetic strategy was refined and optimized, among others, by optimizing choice of functionalizing agents to ensure improved long-term stability of the nanomaterials, allowing, through several controlled synthesis tests, to enhance reproducibility and to minimize both polydispersity and colloidal instability. Specifically, adjustments were made to key synthetic parameters, including reagent concentrations and the molar ratio of gold or silver precursors to stabilizing agents, to obtain a nanoparticle population with a narrower size distribution and greater long-term colloidal stability. These optimizations led to a significant reduction in the polydispersity of the synthesized NPs, resulting in more uniform morphology and greater reproducibility across different synthesis batches. The results demonstrate a reliable synthetic approach, characterized by high colloidal stability and low size dispersion, which are critical features for applications in the biomedical and nanodiagnostic fields.

Although PEGylation is traditionally used to improve colloidal stability and reduce nonspecific interactions, PEG-coated nanoparticles often exhibit an uncontrolled polydispersity and heterogeneous surface properties. This variability can lead to unpredictable interactions with biomolecules and physiological media, ultimately compromising the *in vivo* stability, circulatory behavior, and overall biological performance. For this reason, it is essential to ensure that a well-defined and controlled amount of PEG is bound to the nanoparticle surface. An insufficient or nonuniform PEG coating can promote instability, which can be detected by postsynthesis UV–vis spectra. However, this instability can be ameliorated by the appropriate optimization of the synthesis conditions.

These limitations were observed even when standard literature protocols were applied in this work, resulting in AuNPs-PEG exhibiting consistent UV–vis spectra with broad size distributions and inconsistent colloidal stability. For these reasons, the main effort of the optimization process conducted in this work was dedicated to refining the synthesis of AuNPs-PEG. A systematic series of synthesis trials were conducted in which the molar ratio of the stabilizing PEG ligand to the gold precursor was varied in a controlled manner. The goal was to identify the optimal experimental conditions capable of promoting efficient nucleation and growth of the metal core while obtaining monodisperse, stable, and highly reproducible nanoparticles.

Figure S1, shown in the Supporting Information, shows the UV–vis spectra of AuNPs-PEG synthesized using different molar ratios of PEG-SH to gold. At low concentrations (black) of the stabilizing agent, the plasmon resonance peaks appear broad and poorly defined, indicating the formation of nanoparticles with heterogeneous shapes and sizes, which tend to aggregate, resulting in a broadened plasmon band. As the concentration of PEG-SH increases, the plasmon resonance peaks become sharper and more defined, reflecting the formation of nanoparticles with more uniform size and shape distributions and reduced aggregation (blue and green lines).

Although the protocols are the same for all NPs, the reaction time is different: 2 h for AuNPs-cit-l-cys and AgNPs-cit-l-cys and 20 min for AuNPs-PEG and AgNPs-PEG. The observed variation in the time required for NP formation may be attributed to the distinct nature of the surface capping agents; citrate and l-cysteine exhibit stronger interactions with the gold surface, through electrostatic attraction and thiol, gold coordination, respectively. In contrast, PEG-SH weakly interacts with the gold or silver surface, thereby maintaining greater accessibility of active sites and facilitating a more efficient reduction by NaBH_4_. The formation of nanoparticles is confirmed by UV–vis spectroscopy analysis.

Figure 1a shows the UV–vis comparison spectra of AuNPs-PEG and AgNPs-PEG analyzed in the same concentrations. The maximum absorption peak of AuNPs-PEG (blue) is around 540 nm and around 410 nm for AgNPs-PEG (green). Both metals have good reduction potential, but although gold has it more positively than silver, the peak uptake of AgNPs-PEG appears to be sharper, more evident, and symmetrical, indicating a homogeneous nanoparticle population with uniform size and morphology, reflecting low polydispersity and high colloidal stability. In contrast, the broad and poorly defined peak exhibited by AuNPs-PEG suggests a heterogeneous distribution of particle sizes and shapes, often correlated to aggregation and decreased stability. This will also be confirmed by morphological analysis (conventional TEM (CTEM)) in which AgNPs (Figure 1b) appear to be less polydisperse and less aggregated than AuNPs (Figure 1c). AgNPs-cit-l-cys (Figure 1d, green) behaves similarly to AgNPs-PEG shown above, with maximum absorption around 400 nm and a sharp plasmon resonance peak. In contrast, AuNPs-cit-l-cys (Figure 1d, blue line) shows a red-shifted and broader absorption band, with a plasmon resonance peak around 560 nm. These results reveal differences in particle size distribution and aggregation states. These data are further confirmed by HR-TEM images reported in Figure 1e, showing a higher degree of aggregation for AuNPs-cit-l-cys than that observed in Figure 1b. The morphology of AgNPs-cit-l-cys is reported in Figure 1f, showing spherical shapes and different ramifications.

**Figure 1:**
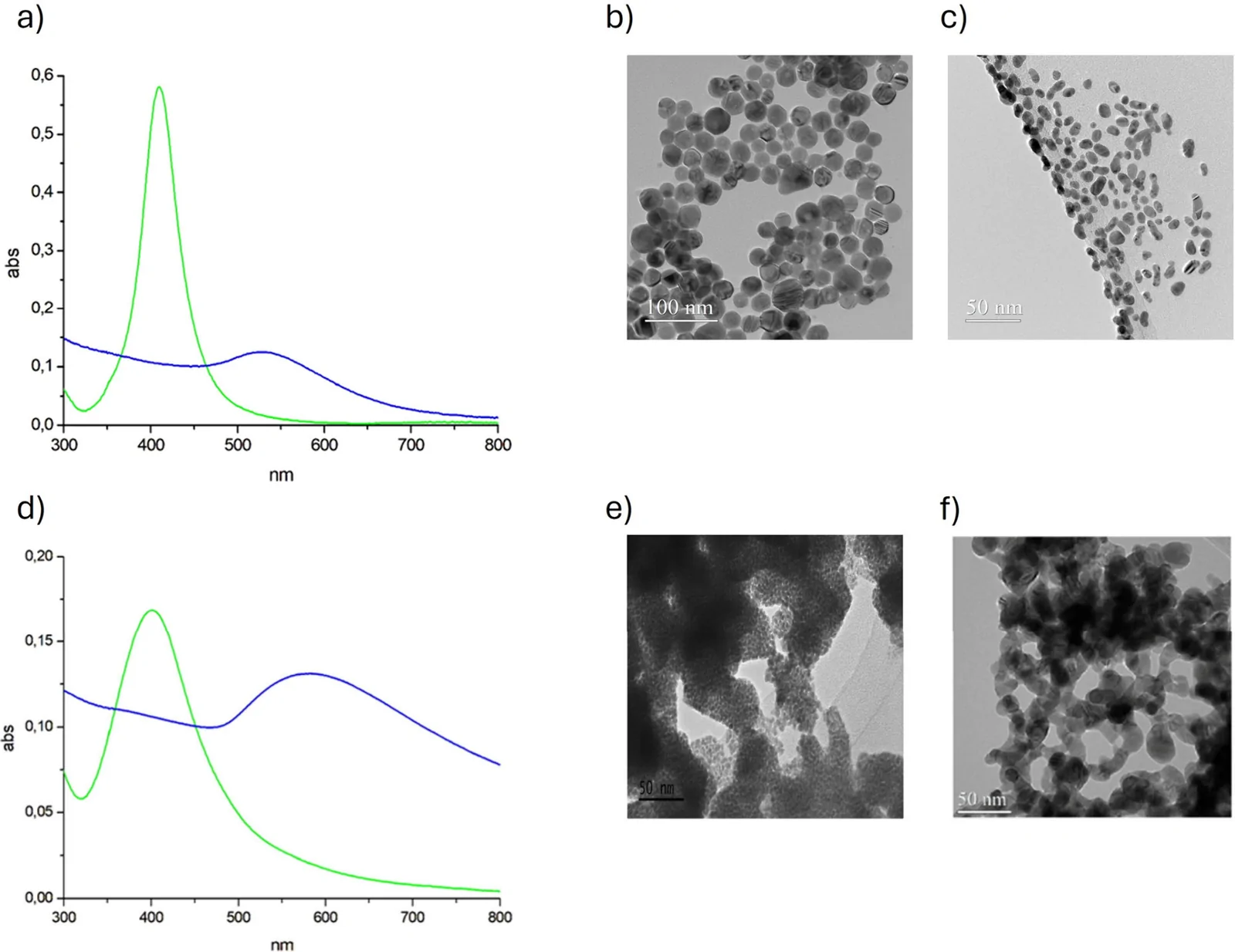
a) UV–vis absorption spectra of AuNPs-PEG (blue line, λ_max_ = 540 nm) and AgNPs-PEG (green line, λ_max_ = 410 nm). b) CTEM image of AgNPs-PEG. c) CTEM image AuNPs-PEG. The average diameter of the AuNPs is 12 ± 2 nm (on 100 NPs) and 30 ± 8 nm for AgNPs (on 100 NPs). d) UV–Vis absorption spectra of AgNPs-cit-l-cys (green line, λ_max_ = 400 nm) and AuNPs-cit-l-cys (blue line, λ_max_ = 560 nm) NPs. e) HR-TEM images of AuNPs-cit-l-cys. f) TEM of AgNPs-cit-l-cys.

UV–Vis spectrum of AuNPs-PEG shows a more intense and defined SPR, indicating a narrower size distribution and a lower tendency to aggregate. These characteristics suggest effective steric stabilization provided by PEG-SH, which prevents direct contact between the nanoparticles and maintains their colloidal dispersion. In contrast, the spectrum of AuNPs-cit-l-cys has a broader and less pronounced plasmonic band, accompanied by a slight red shift in the λ_max_ value. Such broadening is generally associated with greater polydispersity of the system, which may result from the presence of NPs of varying sizes or from partial aggregation phenomena. In this case, the stabilization provided by the citrate–l-cysteine mixture is less effective, probably due to the limited electrostatic protection compared to the more robust steric barrier exerted by PEG-SH. For AgNPs, both stabilization strategies produce well-defined plasmon resonance peaks. However, as also observed for gold NPs, the AuNPs-PEG displays a more intense signal compared with its citrate-l-cysteine analogue, further supporting the enhanced stabilizing efficiency of PEG-SH.

### 3.2 AuNPs and AgNPs Labeled with FITC

Functionalization of the NPs was done with FITC stain that binds to the nanoparticles via the isothiocyanate group.^40^ Functionalizing nanoparticles with a fluorescent dye is particularly valuable for investigating their biological interactions, which is a critical aspect for the development of biomedical applications. In this context, FITC enables the sensitive and reliable detection of nanoparticles within biological systems, facilitating their tracking and spatial localization. The use of FITC allows nanoparticles to be analyzed by user-friendly, relatively easily accessible techniques such as flow cytometry and confocal microscopy, which are essential for elucidating cellular uptake mechanisms and assessing biocompatibility. The NPs-FITC were analyzed by UV–vis absorption spectroscopy: The supernatant was expected to contain the unbound FITC, while the pellet contained FITC-functionalized nanoparticles, as confirmed by plasmonic absorption and fluorescence spectra reported in Figure 2; more in detail, the absorption spectrum of the supernatant shows a signal around 490 nm due to the typical absorption of FITC, while the spectrum of the pellet has 2 peaks respectively related to the plasmonic absorption of NPs at 550 nm for AuNPs and 400 nm for AgNPs, and to FITC molecules. This behavior is clearly observable in AuNPs-cit-l-cys (Figure 2c), where the presence of a second absorption peak is evident. In contrast, in AuNPs-PEG (Figure 2d) the secondary peak is not clearly distinguishable; instead, only an overall increase in absorbance intensity is observed, which may be attributed to the presence of FITC. For AgNPs (Figure 2a,b), a weak shoulder at about 500 nm is observed only for AgNPs-PEG (Figure 2b). Nonetheless, the presence of FITC on all nanoparticle surfaces was unambiguously assessed by other methods, first of all fluorescence emission spectroscopy. Figure 2e-f show the FITC emission signal of AuNPs-cit-l-cys and AgNPs-cit-l-cys, respectively, after being excited at 490 nm. For both, the fluorescence signal was around 515 nm. In the case of AuNPs-cit-l-cys (Figure 2e), an intense and well-defined fluorescence signal is observed, with a maximum peak of approximately 60 au, indicative of efficient grafting of the fluorophore on the NP surface. The high emission intensity suggests that AuNPs promote the stability and emissive efficiency of the FITC. In contrast, in the AgNPs-cit-l-cys spectrum (Figure 2f), the fluorescence intensity is significantly reduced, while maintaining the same emission wavelength.

**Figure 2:**
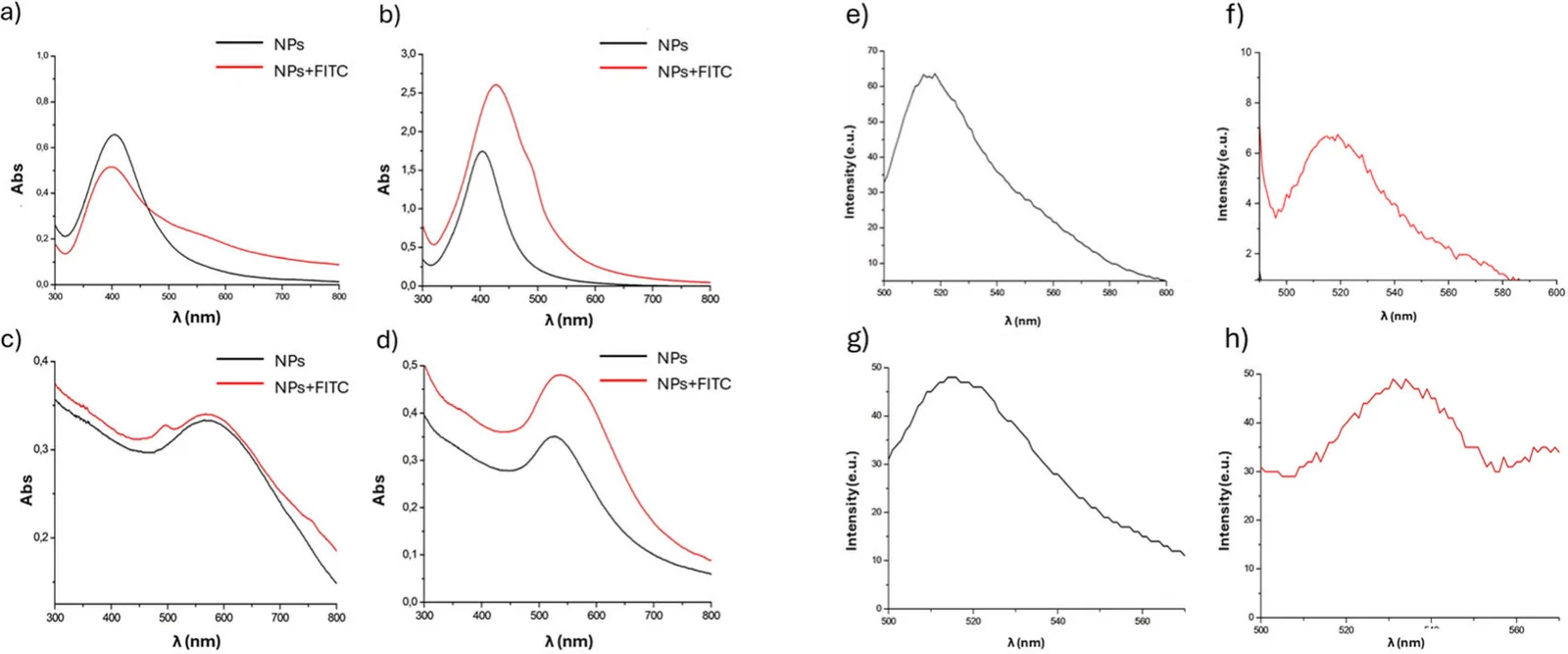
UV–vis absorption spectra of NPs functionalized with FITC. (a) Absorption spectrum of AgNPs-cit-l-cys (black) and AgNPs-cit-l-cys-FITC (red). Absorption peaks have a maximum around 400 nm. (b) Absorption spectrum of AgNPs-PEG (black) and AgNPs-PEG-FITC (red). Absorption peaks have a maximum around 400 nm, and there is evident a slight shoulder around 500 nm. (c) Absorption spectrum of AuNPs-cit-l-cys (black) and AuNPs-cit-l-cys-FITC (red) showing an increase in absorption in the 490–500 nm region, indicative of FITC. (d) Absorption spectrum of AuNPs-PEG (black) and AuNPs-PEG-FITC (red). Absorption peaks have a maximum around 550 nm. e-f) Fluorescence emission spectrum of FITC recorded in solution of functionalized NPs. e) represents the emission spectrum of AuNPs-cit-l-cys-FITC; f) emission spectrum of AgNPs-cit-l-cys-FITC. g) Emission spectrum of AuNPs-PEG-FITC. h) Emission spectrum of AgNPs-PEG-FITC. The emission maximum is observed around 515 nm, a wavelength characteristic of FITC.

### 3.3 Z Potential and DLS Size Analysis

For all synthesized NPs (AuNPs-cit-l-cys; AuNPs-PEG; AgNPs-cit-l-cys; AgNPs-PEG), DLS analyses were performed before and after FITC labeling with the aim to study the modifications in size and zeta potential (ζ). The size of NPs was evaluated by measuring the Hydrodynamic Radius (⟨2*R*_H_⟩) through DLS analysis. 2RH is defined as the radius of the hypothetical hard sphere that diffuses with the same velocity as the particles analyzed under DLS. Thus, RH is a hypothetical measurement since such hard spheres rarely exist in colloidal dispersions.

DLS analysis (Figure 3a-d) shows the effect of FITC functionalization on the ⟨2*R*_H_⟩ of the NPs. For all the samples, the polydispersity index is less than 1, an indicator of good polydispersity. The reported size values refer to the *main intensity peak* (the average of 3 measurements) rather than the *Z-average*. For AuNPs-cit-l-cys (Figure 3 a), the distribution remains monomodal and centered at199 nm, with a slight increase in ⟨2*R*_H_⟩ after conjugation with FITC ⟨2*R*_H_⟩ = 240 nm, indicating successful functionalization without the formation of significant aggregates. In the case of AgNPs-cit-l-cys (black line, Figure 3 b), a rather broad distribution can be observed, with the main peak at 150 nm and a tail toward smaller sizes. After functionalization with FITC (red curve, Figure 3b), the distribution shifts toward slightly larger sizes and becomes more irregular, with a small secondary peak toward smaller sizes. Therefore, the average increase in ⟨2*R*_H_⟩ confirms the functionalization, and the presence of a second small peak shows the formation of a smaller subpopulation. The greater irregularity may indicate interactions that modify colloidal stability. AuNPs-PEG (Figure 3 c) displays a clear transition from a monodisperse population (∼100 nm) to a bimodal distribution, with a second peak at larger sizes (∼446 nm), indicating the formation of aggregates following FITC grafting. In contrast, AgNPs-PEG (Figure 3 d) shows an apparent decrease in average size after the addition of FITC, which could be attributed to a change in the scattering profile due to the presence of the fluorophore, rather than a real reduction in size. Generally, a change in ⟨2*R*_H_⟩ is observed after functionalization, confirming the interaction between FITC and the NPs surface, although the extent of the effect depends on the ligand already stabilizing the NPs surface (either citrate - l-cysteine or PEG-SH) and the selected metal (Au or Ag). Among the analyzed samples, AuNPs-cit-l-cys shows the most stable and unimodal distribution, with only a slight increase in ⟨2*R*_H_ ⟩ after conjugation with FITC, indicating that functionalization did not significantly compromise their colloidal stability. In contrast, Ag and AuNPs stabilized with PEG-SH exhibit broader or bimodal distributions after the addition of FITC, suggesting greater heterogeneity or a partial tendency to aggregate.

**Figure 3:**
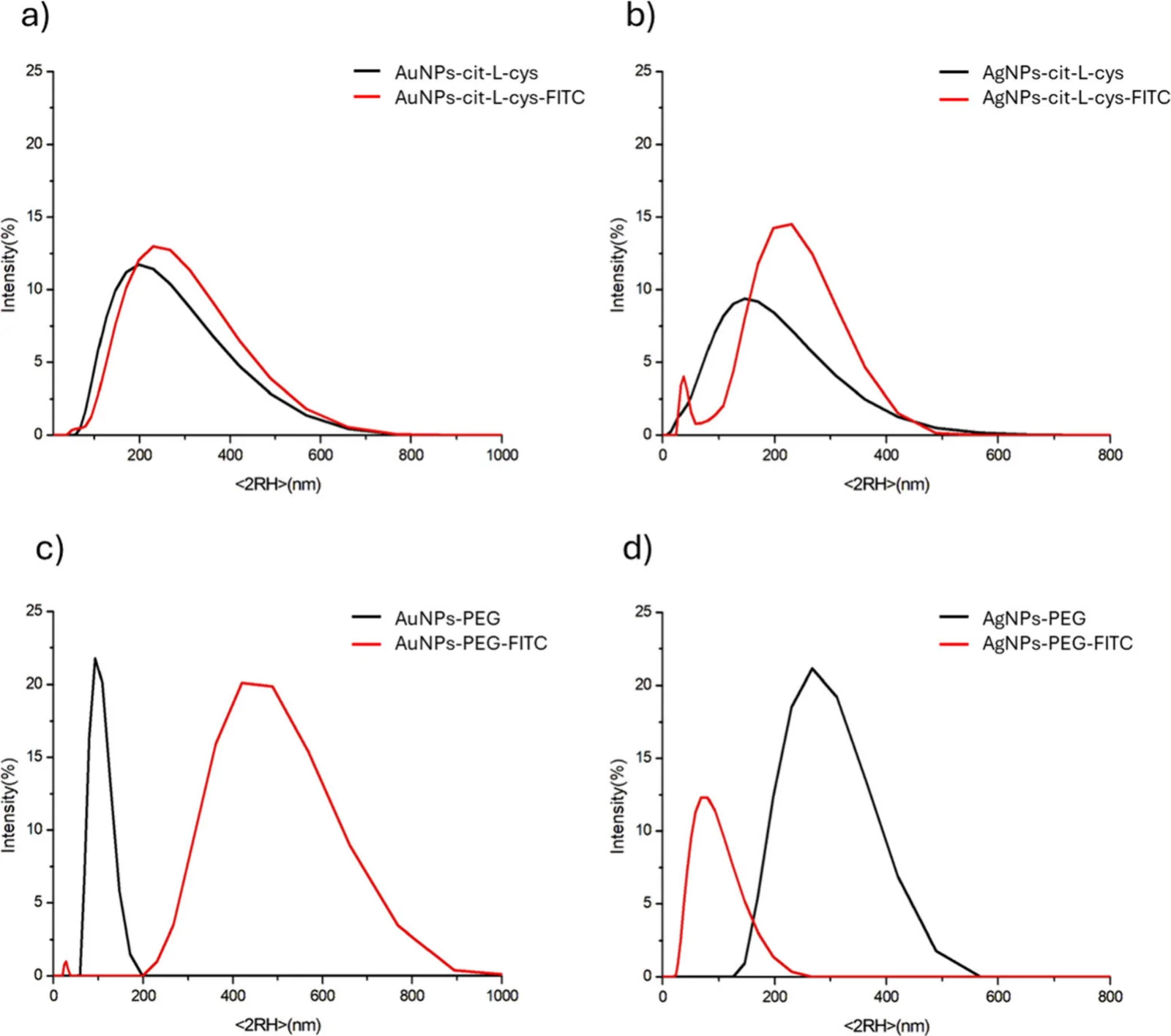
DLS intensity distributions of Au and Ag nanoparticles before (black) and after (red) functionalization with FITC. (a) AuNPs-cit-l-cys (199 ± 24 nm -242 ± 21) and (b) AgNPs-cit-l-cys (141 ± 31 nm – 219 ± 18). (c) AuNPs-PEG (102 ± 7 nm -446 ± 73 nm). (d) AuNPs-PEG (267 nm -76 ± 6 nm).

To assess the colloidal stability of the NPs, the ζ potential analysis was performed. This measurement provides critical insights into the electrostatic interactions governing NPs dispersion stability and offers an indication of their potential cytotoxicity toward biological membranes. In an ionic solution, NPs with a net charge develop a layer of counterions strongly bound to their surface. Surrounding this is a second, diffuse outer layer composed of loosely associated ions. Together, these two layers form the electrical double layer (EDL). When the NPs move, due to Brownian motion, a distinction arises between the ions in the diffuse layer that move with the NPs and those that remain associated with the bulk dispersant. The electrostatic potential at the boundary is termed the ζ, which is closely related to the surface charge of the NPs. In ζ measurements, an external electric field is applied to the sample, inducing NPs motion. This movement is analyzed using ζ and is then calculated using Henry’s equation.^41,42^ NPs with a ζ potential between −10 and +10 mV are considered approximately neutral, while NPs with ζ above +30 mV or below −30 mV are considered strongly cationic and anionic, respectively. In the external range −30 to +30 mV the colloidal suspension is stable. Several studies^43,44^ have shown that a positive ζ is associated with greater NPs toxicity than a negative one because it is more attracted to the negative charges on cell membranes. It is important to note that this is not the only reference value for establishing toxicity.^45^

Figure 4 shows the ζ distributions of Au and AgNPs, with all different stabilizations, before and after functionalization with FITC. The ζ of AuNPs-cit-l-cys (Figure 4 a) was found to have a value of −40 ± 0.5 mV before functionalization and −27 mV after functionalization. This is a good value as it indicates good electrostatic repulsion between the nanoparticles and thus a reduced aggregation phenomena. The decrease in negativity is due to the introduction of groups that partially shield the surface charges. In the case of AgNPs-cit-l-cys (Figure 4 b), the ζ potential shows a more marked variation: functionalization with FITC results in a shift toward positive values, from −38 ± 1.6 mV (in black) to 19 ± 5 mV (in red), indicating a significant change in surface chemistry and a possible reduction in colloidal stability. This behavior can be attributed to the electrostatic interaction between the negative charges of citrate - l-cysteine and the positive groups of FITC on different NPs. For AuNPs-PEG and AgNPs-PEG (Figure 4 c and d), the ζ trend is similar. All samples show an increase in ζ potential toward less negative values after functionalization with FITC. In gold nanoparticles the variation is minimal; on the contrary, for AgNPs-PEG the variation is more marked. This indicates a greater possibility of aggregation and therefore less colloidal stability. Therefore, although FITC modifies the surface of both Au- and Ag-based nanoparticles, the effect is more consistent on AgNPs, consistent with DLS observations that show more pronounced variations in size distribution for silver-based samples.

**Figure 4:**
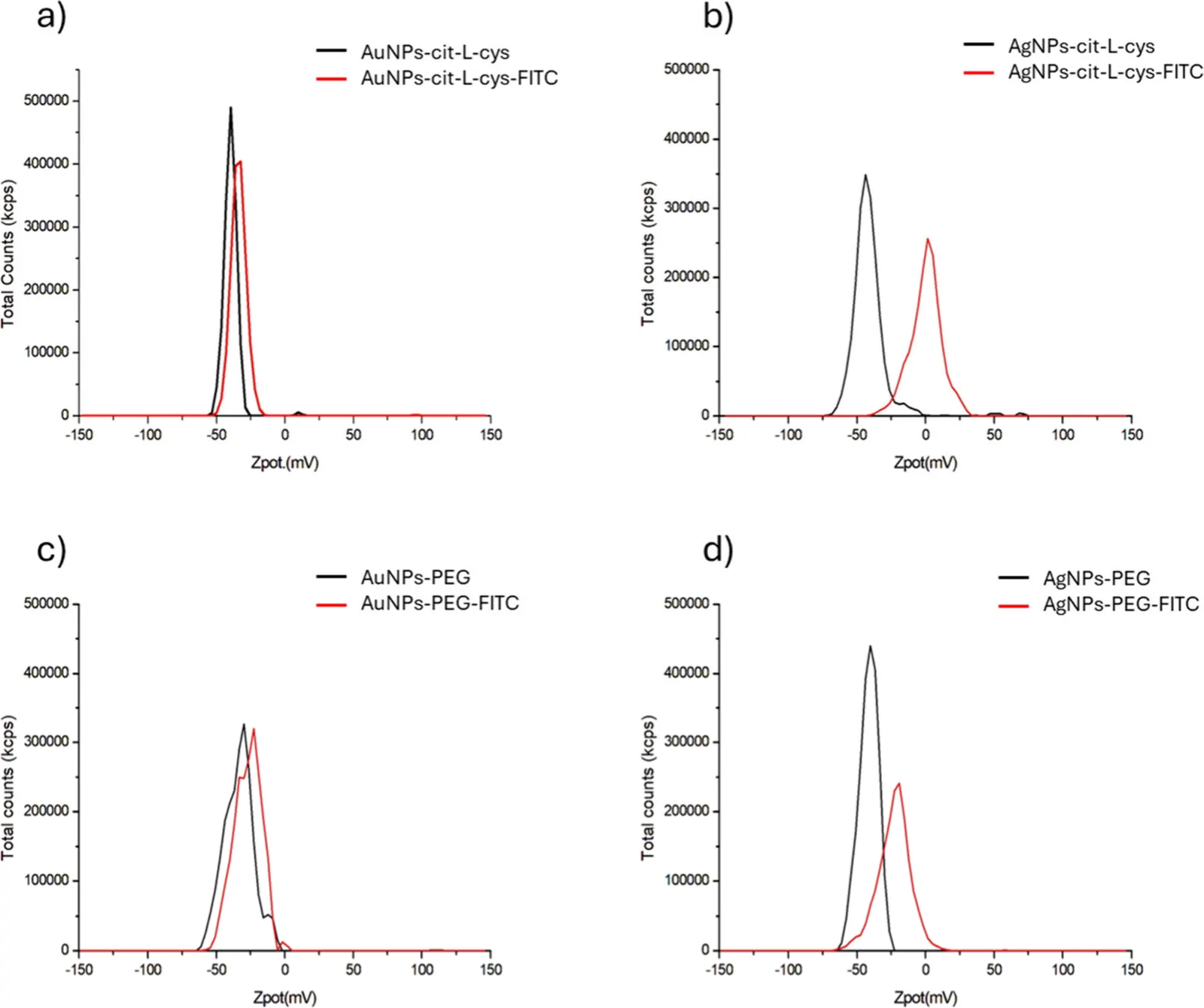
Distributions of ζ of AuNPs and AgNPs before (black lines) and after (red lines) functionalization with FITC. a) AuNPs-cit-l-cys and AuNPs-cit-l-cys-FITC (ζ = −40 ± 0,5 and −27 ± 0,7 mV); (b) AgNPs-cit-l-cys and AgNPs-cit-l-cys-FITC (ζ = −38 ± 1.6 and +19 ± 5 mV); (c) AuNPs-PEG and AuNPs-PEG-FITC (ζ = −32 ± 2.4 and −27 ± 2.3 mV); (d) AgNPs-PEG and AgNPs-PEG-FITC (ζ = −41,34 ± 0.65 and −22 ± 1 mV).

Among the analyzed systems, AuNPs-cit-l-cys displays the best colloidal stability: the DLS distribution remains monomodal and essentially unchanged after functionalization with FITC, and the ζ value undergoes only a slight reduction in absolute value, consistent with partial charge screening without inducing significant aggregation.

For the AuNPs-cit-l-cys, DLS measurements in RPMI supplemented with 5% human serum and at two different temperatures, 25 and 37 °C, were conducted (Figure S2 in the Supporting Information). From these investigations, an increase in particle size was observed when the nanoparticles were resuspended in RPMI supplemented with 5% human serum compared to water, particularly at 37 °C compared to 25 °C. These results are consistent with previous studies, supporting the hypothesis that the formation of a *biocorona*, following interaction with biological components, leads to an increase in the hydrodynamic radius of the nanoparticles. The increase in hydrodynamic diameter observed at 37 °C compared to 25 °C can be attributed to the increased mobility of biomolecules in the *biocorona* and an increase in Brownian motion in general. These effects contribute to an apparent enlargement of the hydrodynamic radius in biological environments and do not affect the efficiency of the drug delivery system.^29,42^

### 3.4 Flow Cytometric Analysis

As previously mentioned, the efficacy of FITC-functionalized nanoparticles was further evaluated by using flow cytometry in view of applications in biomedicine. This technique was used to confirm the presence and detectability of the fluorescent dye on the nanoparticles, providing further validation of the functionalization process. Furthermore, since flow cytometry is a key analytical tool for studying nanoparticle-cell interactions from a biological perspective, the detection of the FITC signal demonstrates the suitability of the functionalized nanoparticles for future studies of NP-cell interactions.

On the basis of physicochemical analyses, gold nanoparticles (either stabilized with citrate-l-cysteine or PEG-SH) were selected as the most promising platform for further investigation through this technique, due to their superior stability and biocompatibility compared to AgNPs, in line with the findings of previous literature.^46^

An initial analysis was performed using flow cytometry to confirm the presence of FITC on the NP surface (Figure 5a,b). The figure shows the median fluorescence intensity (MFI) and the frequency (%) of the signal detected in the FITC channel for AuNPs-PEG (a) and AuNPs-cit-l-cys (b) compared with the same nanoparticles functionalized with FITC. The red peaks represent the negative control, corresponding to the signal generated by AuNPs before the FITC grafting. In contrast, the light blue and blue peaks are collected on the AuNPs functionalized with FITC. Both the frequency and MFI in the FITC channel increase markedly. This provides clear evidence of successful FITC functionalization of the NPs and demonstrates the potential of this technique for evaluating NP fluorescent behavior. The same analysis was also applied to compare the functionalization efficiency between the two AuNPs stabilized with citrate-l-cysteine or PEG-SH. In a second set of experiments, reported in Figure 5 c, the MFI of FITC-functionalized AuNPs-PEG-FITC and AuNPs-cit-l-cys-FITC is directly compared. The data indicate a significantly lower functionalization efficiency of AuNPs-PEG with FITC compared to AuNPs-cit-l-cys. Specifically, the FITC-positive signal for AuNPs-PEG-FITC is much less pronounced (MFI:8973), while AuNPs-cit-l-cys-FITC exhibits a markedly higher MFI (21330), highlighting their superior capacity for FITC conjugation. For these reasons, combined with the results obtained from other chemical-physical analyses described below, AuNPs-cit-l-cys was chosen as the best candidate for further biological interaction studies.

**Figure 5:**
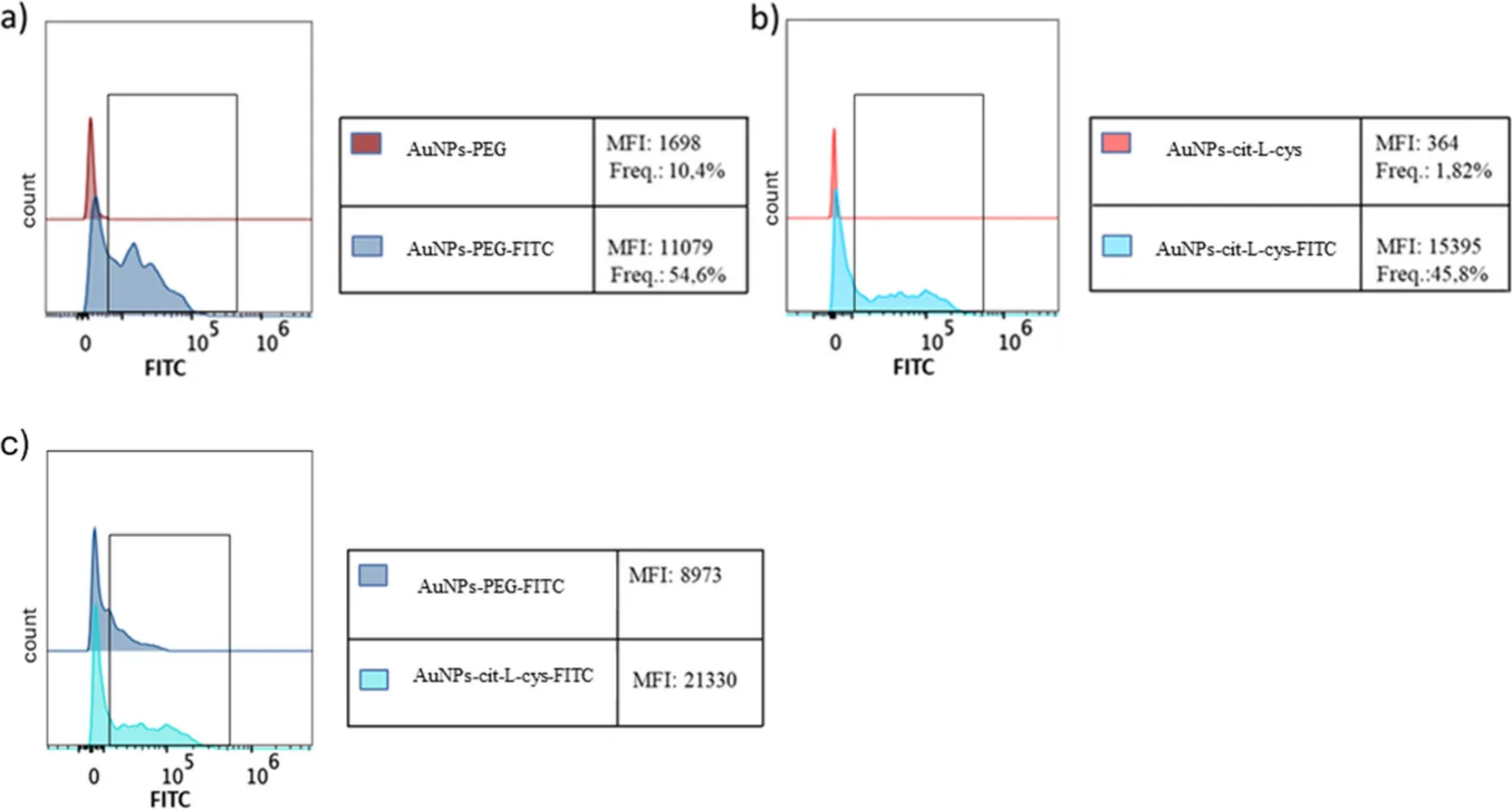
Flow cytometry investigations reported the fluorescence signal detected on the FITC channel for: a) AuNPs-PEG. The red peaks represent the negative signal corresponding to nanoparticles before functionalization with FITC (MFI:1698; Freq: 10,4%). The blue signals, composed of both negative and positive populations, correspond to FITC-functionalized NPs (MFI: 11079; Freq: 54,6%). b) AuNPs-cit-l-cys. The red peaks represent the negative signal corresponding to nanoparticles before FITC grafting (MFI:364; Freq: 1.82%). The blue signals, composed of both negative and positive populations, correspond to FITC-functionalized NPs (MFI: 15395; Freq: 45.8%). c) Comparison of the FITC signal MFI between FITC-functionalized AuNPs-PEG-FITC (blue) and AuNPs-cit-l-cys-FITC (light blue). The blue peak shows a weak positive signal with an MFI of 8973. In contrast, the light blue peak, clearly visible in the positive region, has a higher MFI of 21330. These results indicate a greater efficiency of FITC functionalization for AuNPs-cit-l-cys.

### 3.5 SR-XPS Analysis of Molecular and Electronic Structure at the NPs/Ligands Interface

Synchrotron radiation-induced X-ray photoelectron spectroscopy (SR-XPS) measurements were applied to investigate the functionalization of AuNPs and AgNPs with PEG, l-cysteine, citrate, and FITC and their structural stability and chemical properties. Experiments were carried out on AuNPs-cit-l-cys, AuNPs-Cit-l-Cys-FITC, AuNPs-PEG, AuNPs-PEG-FITC, AgNPs-cit-l-cys, AgNPs-cit-l-cys-FITC, AgNPs-PEG, AgNPs-PEG-FITC, and pristine PEG-SH samples, collecting spectra at C 1s, N 1s, S 2p, and Au 4f or Ag 3d core levels. A table summarizing the main data analysis results (BE (eV), fwhm (eV), relative intensity values, and proposed signal assignments) can be found in the Supporting Information as Table S1.

C 1s core level spectra for AuNPs-cit-l-cys, AuNPs-cit-l-cys-FITC, AuNPs-PEG, AuNPs-PEG-FITC, and the analogous AgNPs-based samples, as well as pristine PEG, are reported in Figure S3 in the Supporting Information. All spectra of nanoparticles stabilized by citrate-l-cysteine display components associated with C–C, C–S, C–N, C–O, C=O, and COOH signals,^47,48^ confirming the presence of the two ligands. C 1s spectra collected on PEG-SH-containing samples have a similar shape, and by applying a peak-fitting procedure, it was possible to point out C–C, C–S, C–O, and COOH contributions in excellent agreement with the C 1s spectrum collected on pristine PEG (Figure S3).

The most indicative signals for the investigation of all AuNP and AgNP functionalization steps are N 1s and S 2p, since S 2p signal components are indicative for the interaction at the metal NP surface/thiol molecule interface, and N 1s spectral components will allow us to ascertain the successful grafting of FITC through the SCN-related nitrogen spectral component. N 1s core level spectra, collected for AuNPs-cit-l-cys-FITC, AuNPs-PEG-FITC, AgNPs-cit-l-cys-FITC, and AgNPs-PEG-FITC, are all composited; FITC-containing nanoparticles (Figure 6 a-d) show spectral components ascribed to -N=C=S functional groups, amines (RNH_2_) in agreement with the structure of l-cysteine and FITC, and positively charged nitrogen atoms whose formation was expected as previously reported.^49^ The low BE signal in AuNPs-cit-l-cys and AgNPs-cit-l-cys (Table 1) is probably due to electrostatic interaction between the l-cysteine amine group and Ti of the substrate surfaces used for sample deposition.^50^

**Figure 6:**
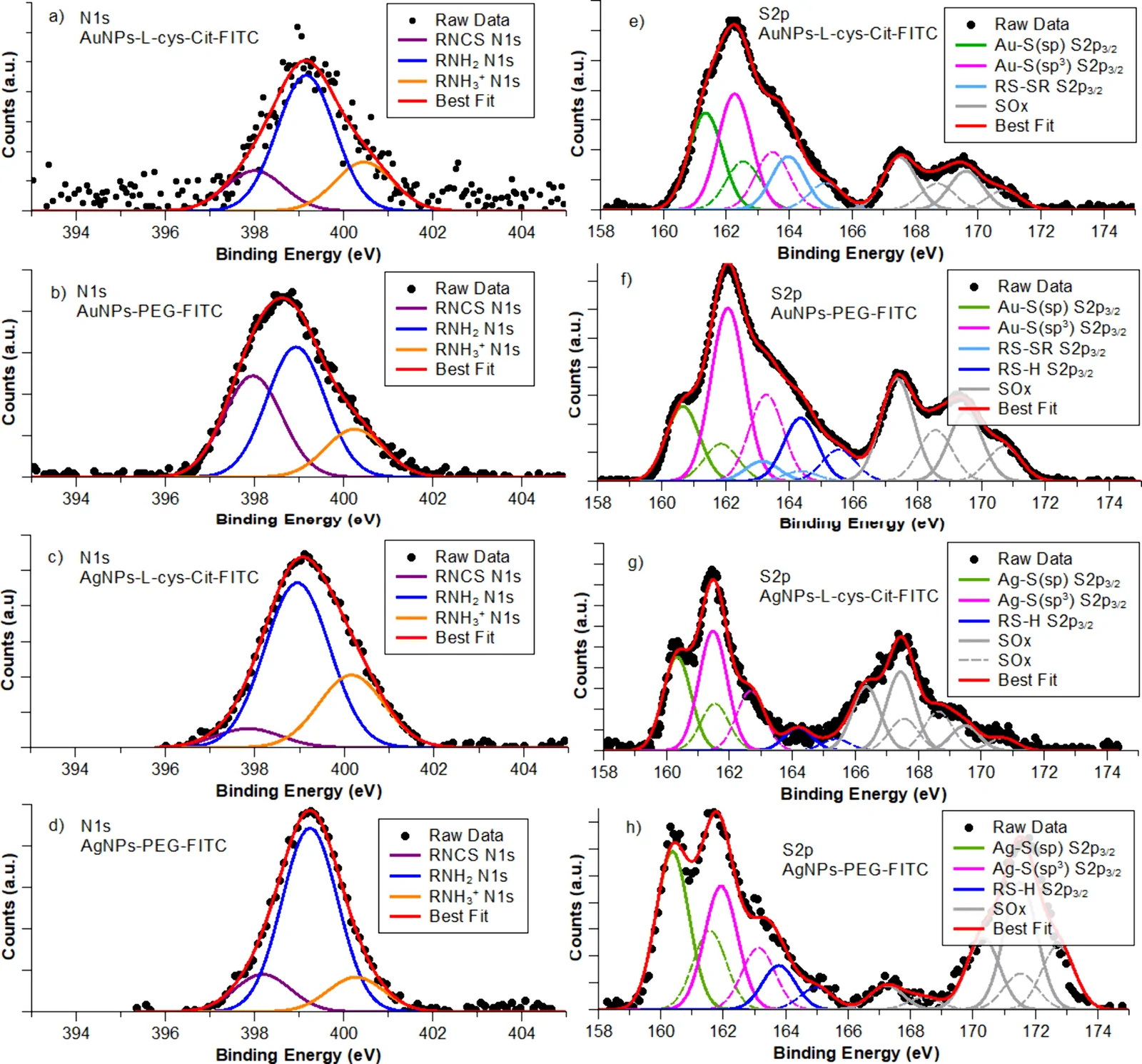
N 1s core-level spectra collected on a) AuNPs-cit-l-cys-FITC, b) AuNPs-PEG-FITC, c) AuNPs-cit-l-cys-FITC, d) AgNPs-PEG-FITC; S 2p core-level spectra of e) AuNPs-cit-l-cys-FITC, f) AuNPs-PEG-FITC, g) AuNPs-cit-l-cys-FITC, h) AgNPs-PEG-FITC.

The S 2p spectra collected on AuNPs-cit-l-cys-FITC, AuNPs-PEG-FITC, AgNPs-cit-l-cys-FITC, and AgNPs-PEG-FITC are reported in Figure 6 d–g. In particular, the S 2p core-level spectra of the four samples show several signals. First, in both AuNPs and AgNPs, at low BE, two peaks related to the presence of M-S with different hybridizations (sp and sp^3^) are noted, evidencing the effective bond between gold or silver and sulfur atoms. At higher binding energies, signals related to disulfides (S–S) and physisorbed thiols (RS-H) are also found, as already observed in previous works.^25,51^ The spectral components located at higher BE (BE > 165 eV) are attributed to the oxidized S atoms^45^ and can be also observed in small amounts in the pristine PEG-SH reference (Figure S4). It is noteworthy that very similar spectral shapes are observed for AuNPs-Lcys-cit, AuNPs-PEG, AgNPs-Lcys-cit, and AgNPs-PEG spectra, reported in Figure S4 in the Supporting Information.

Finally, Au 4f or Ag 3d signals fully confirm the thiol interaction with the metal nanoparticles surface; both Au 4f and Ag 3d spectra (Figure S5 a–d) of NPs functionalized with FITC are asymmetric at a high BE, indicating the presence of at least two different kinds of noble metal atoms: the signal at lower BE values (Au 4f_7/2_ = 83.8 eV, Ag 3d_5/2_ = 367.6 eV) is assigned to metallic gold and respectively silver atoms of the nanoparticle core, while the low-intensity (about 10% of the whole signal) spin–orbit pair at a higher BE (Au 4f_7/2_ = 84.5 eV, Ag 3d_5/2_ = 368.1 eV) is caused by positively charged metal atoms at the NP surface, interacting with the thiol end-group of l-cysteine and PEG-SH.^52^

It is interesting that, comparing the un- and functionalized samples with FITC, S 2p and N 1s core level spectra of the AuNPs and AgNPs do not differ significantly. This aspect is not surprising given the low amount of bonded FITC relative to l-cysteine for both S and N atoms and PEG-SH for S atoms. The effectiveness of FITC functionalization can be confirmed by noting the percentages of C–N and C–S bonds that increase when FITC functionalizes the MNPs. In particular, this is evident in the C 1s components of the AuNPs-cit-l-cys-FITC reported in Table S1. Despite the incertitude in XPS semiquantitative analysis being about 5% (as a best-case scenario^53^), the observed trend is supported by the fluorescence spectra and flow cytometry experiments that allowed us to unambiguously confirm FITC presence in all functionalized samples. As for the FITC/nanoparticle interaction mechanism, the energy resolution of SR-XPS experiments does not allow to specifically point out modifications in N 1s and/or S 2p spectra allowing to exclude a direct interaction between the SCN moiety and the metal surfaces; however, the semiquantitative analysis reported in the third column of Table S1 indicates a stable percentage of S 2p atoms involved in RS-Metal covalent bond (sum of sp and sp^3^ hybridized S) in all nanoparticles functionalized with FITC with respect to the pristine NPs, suggesting that a direct binding to the metal surface is not occurring. This is also supported by NEXAFS C and N K-edge spectra described in the following paragraph and by the literature reporting, for NPs already stabilized by appropriated ligands, FITC-SCN physisorption^40^ or chemisorption through the formation of a covalent bond between the amino groups of l-cysteine and the isothiocyanate groups.^54^

### 3.6 NEXAFS Analysis of the Molecular Structure through FITC Grafting

The C K edge NEXAFS spectra of functionalized nanoparticles AuNPs-cit-l-cys-FITC and AuNPs-PEG-FITC are shown in Figure 7; the spectrum of pristine FITC is also shown for comparison.

**Figure 7:**
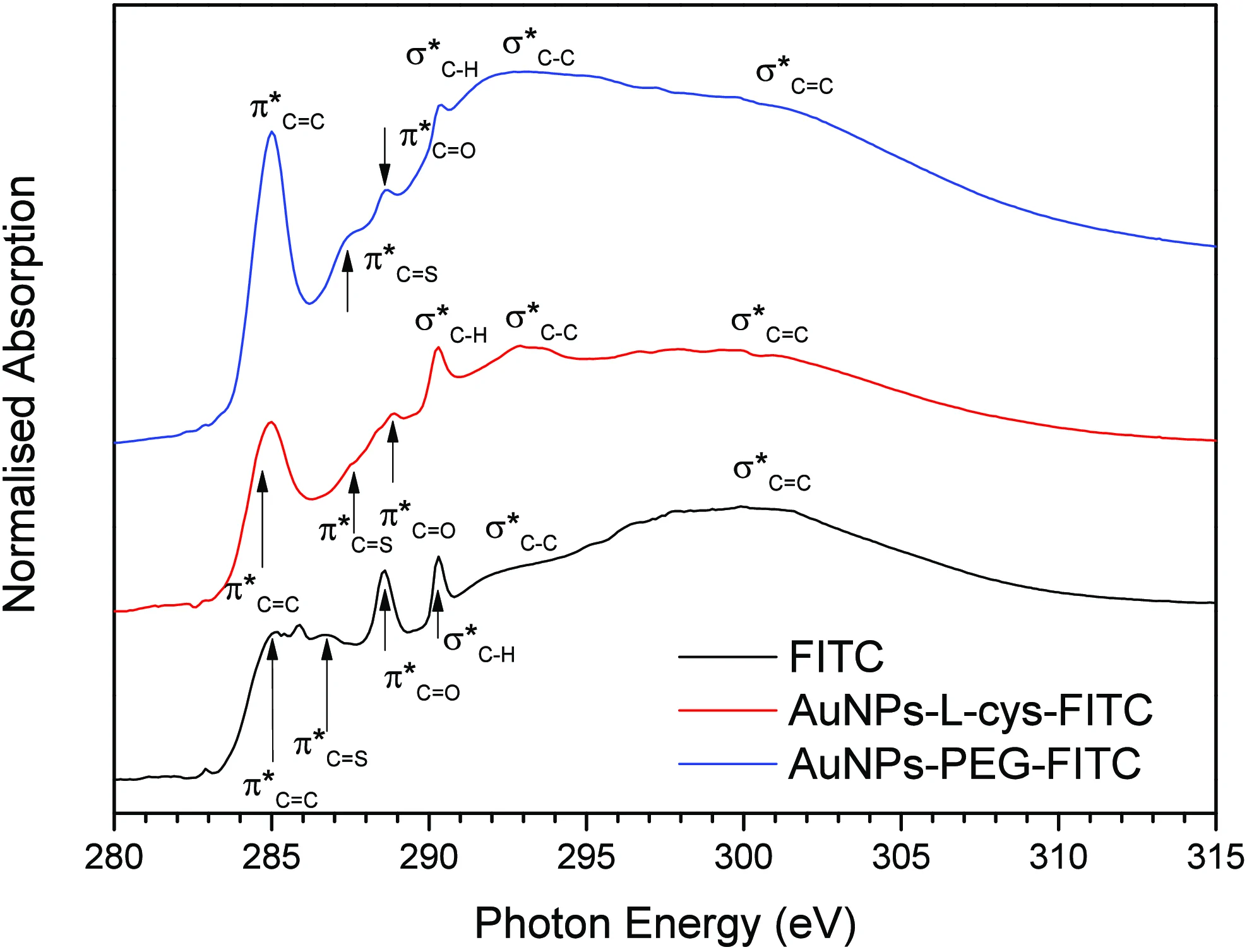
C K edge NEXAFS spectra of FTIC (black line), AuNPs-cit-l-cys-FITC (red line), and AuNPs-PEG-FITC (blue line).

The pre-edge region of the C K edge spectrum of pristine FITC is dominated by 1s→π* transitions arising from the aromatic rings (π*_C=C_, located at 285.0 eV, with a second component at 285.8 eV), from the thiocyanate moiety (π*_C=S_, 286.7 eV), and from the C=O bonds of the carboxyl function (π*_C=O_, 288.8 eV); near the edge, the peak at 290.4 eV can be assigned to transitions related to the C–H σ bond. Above the edge broad σ* resonances detected at about 292 and about 300 eV are assigned to σ* resonances related to singly and doubly bonded caron atoms. Peak positions and relative assignments are in agreement with the results obtained on similar molecules.^31–33^

In the spectra of the functionalized NPSs (AuNPs-cit-l-cys-FITC and AuNPs-PEG-FITC), the peaks related to the FITC moiety are clearly visible at the same photon energy, proving the successful immobilization of FITC on the NP surface. The resonance related to the isothiocyanate function appears slightly shifted due to the reaction of the NCS group with the amine functions forming thiourea.^54^

The N K edge spectra of pristine FITC and of functionalized nanoparticles AuNPs-cit-l-cys-FITC and AuNPs-PEG-FITC are shown in Figure 8.

**Figure 8:**
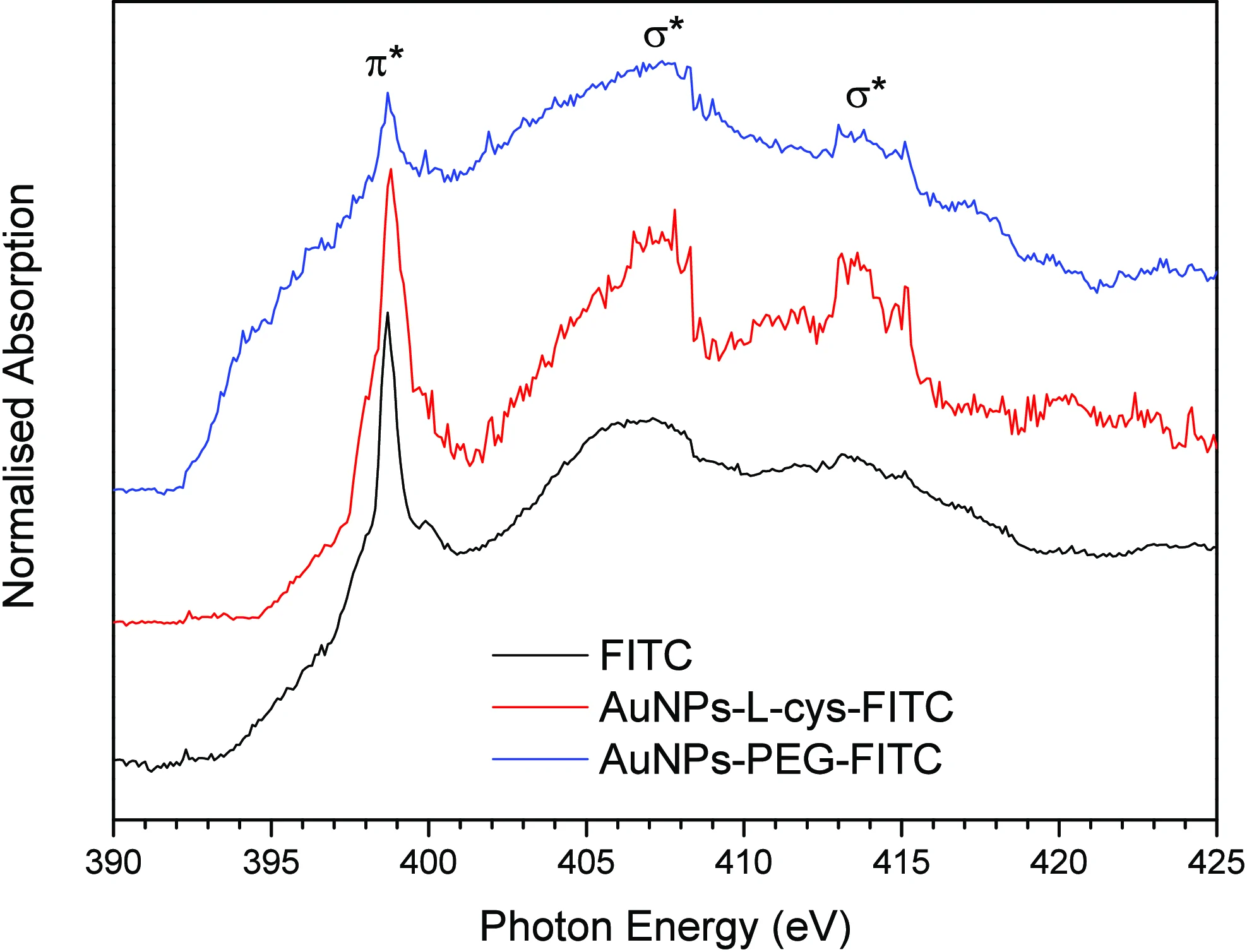
N K edge NEXAFS spectra of FTIC (black line), AuNPs-cit-l-cys-FITC (red line), and AuNPs-PEG-FITC (blue line).

The pre-edge region on the N K edge spectrum of FITC is dominated by the π* resonance related to the isothiocyanate moiety and located at 398.7 eV as reported in^32,33^ above the edge two σ* resonances which are located at 407 and 413 eV. The same resonances are clearly visible in the spectra of the functionalized nanoparticles (AuNPs-cit-l-cys-FITC and AuNPs-PEG-FITC), yielding a further proof of the successful immobilization of FITC on the NPs surface. The π* resonance, which was related to the isothiocyanate moiety, is still detectable, though shifted to slightly higher photon energy (about 0.2 eV), due to conjugation between the C–N and C=S bonds of the thiourea group.

### 3.7 FT-IR- RAIRS Spectroscopy: Functional Groups

The FTIR spectra recorded in the 1900–750 cm^–1^ region, where the most significant bands are located, for FTIC, AuNPs-cit-l-cys-FITC, and AuNPs-PEG-FITC, are shown in Figure 9; peak positions and assignment for the overall FTIR spectra (4000–400 cm^–1^ range) are shown in Table S2.

**Figure 9:**
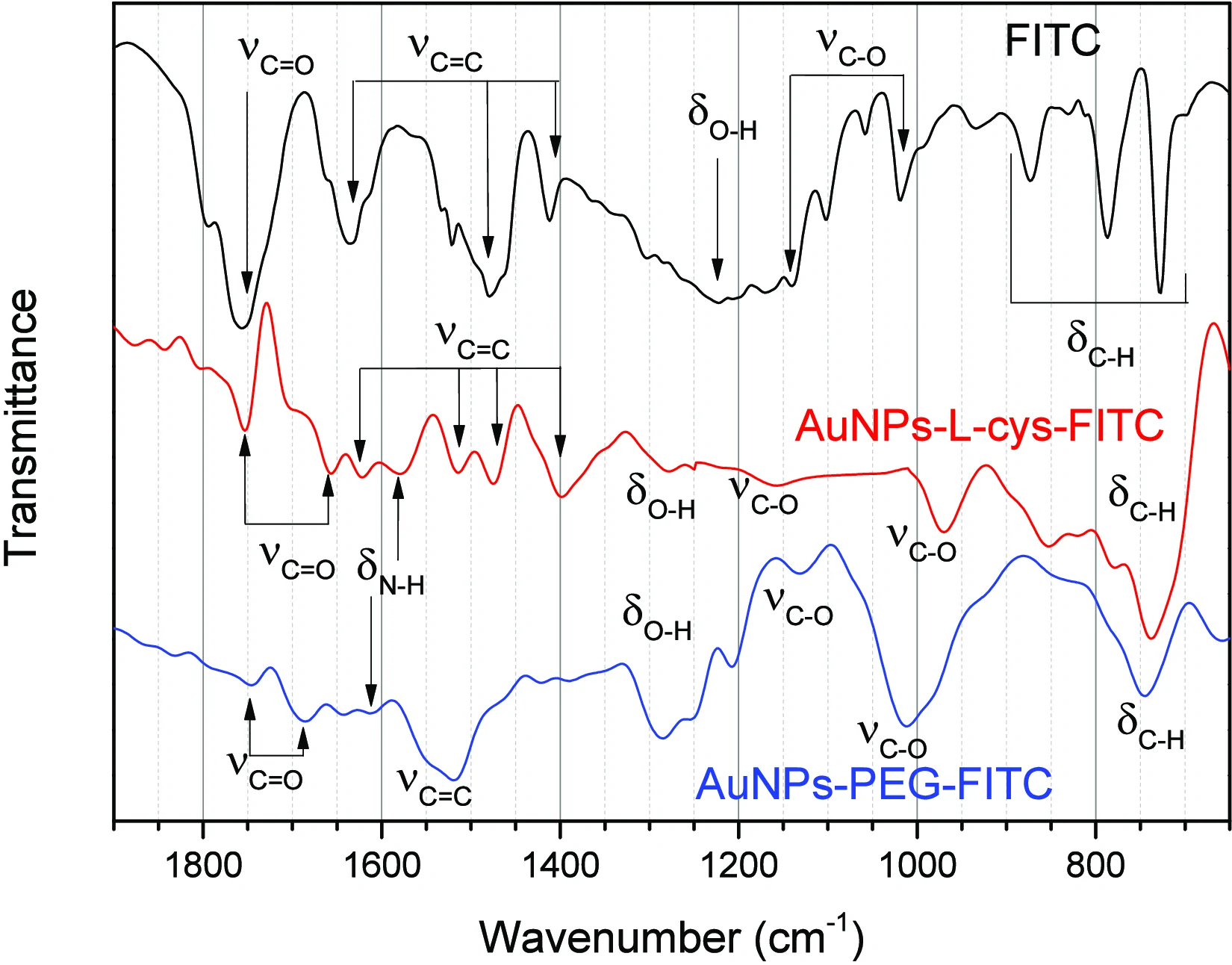
FTIR spectra of FTIC (black line), AuNPs-cit-l-cys-FITC (red line), and AuNPs-PEG-FITC (blue line) in the 1900–750 cm^–1^ region.

In the FTIR spectrum of pristine FITC, the main peaks detected in the 4000–2000 cm^–1^ region can be assigned to the stretching vibration of the O–H and C–H bonds and to the stretching of the isothiocyanate function.

In the 1900–650 cm^–1^ range, the main peaks detected can be assigned the vibrational modes of double (ν_C=O_) and single (ν_C–O_) bonds of the ester function, to the C=C stretching (ν_C=C_) and C–H bending (δ_C–H_) of the aromatic rings, and finally to the to the C–O stretching (ν_C–O_) and O–H bending (δ_O–H_) of the phenol functions.^55^

All the peaks detected in the 1900–650 cm^–1^ region of the FITC spectrum are still clearly visible, though broadened and reduced in intensity, in the corresponding spectra of functionalized NPS (AuNPs-cit-l-cys-FITC and AuNPs-PEG-FITC), supporting the evidence of the effective FITC immobilization on the NPS surface.

The peaks related to isothiocyanate cannot be detected in the same position in the spectra of the functionalized NPs. New vibrational modes related to the stretching (see table S2) and bending (δ_N–H_) of the N–H bonds appear when the isothiocyanate group is converted to thiourea upon FITC immobilization on the NPs surface.

Other peaks detected in the spectra of the functionalized nanoparticles might be related to the organic molecules anchored to the NP surface before functionalization with FITC; this effect is due to the higher sampling depth of RAIRS spectroscopy compared with more surface-sensitive techniques such as XPS and NEXAFS.

For instance, both AuNPs-cit-l-cys-FITC and AuNPs-PEG-FITC show a second C=O stretching band at lower wavenumber, probably due to the carboxylate groups of l-cysteine and citrate; the C=S bond of the thiourea group might also contribute to this peak, though its intensity is expected to be lower compared to C=O stretching modes, due to reduced dipole moment. Vibrations due to the ammonium cation of l-cysteine might contribute to the intensity of bending (δ_C–H_) and stretching (Table S2) modes of the N–H bonds. In the spectrum of AuNPs-PEG-FITC the increase in intensity of the C–O stretching band at 1020 cm^–1^ is probably due to the superimposition with the corresponding peak of PEG.

### 3.8 Biological Tests

From among the NPs investigated and discussed in this work, we selected those deemed most promising both for the molecular structure stability of the stabilizing agents and FITC, as assessed by the spectroscopic SR-XPS, NEXAFS, and FTIR investigation, and for their high capacity to interact with the fluorescent dye. In particular, the flow cytometry analysis of the fluorescence signal, shown in Figure 5, clearly highlights how AuNPs-cit-l-cys exhibits significantly higher intensity in the FITC channel. The data indicate a more efficient presence of FITC compared to the other formulations considered. In addition to confirming the surface functionalization properties of the nanoparticles, the test also identified flow cytometry as a reliable and sensitive technique for studying the behavior of fluorescent NPs. Based on these preliminary results, it was possible to initiate an initial characterization of the interactions between nanoparticles and cellular systems. To this end, PBM cells were selected, as they represent one of the first cell types to come into contact with the nanoparticles following their introduction into the body. Cell viability data were obtained by flow cytometry analysis using the LIVE/DEAD, Dead Cell Stain kit. In cells with compromised membranes, the dye reacts with free amines both on the cell surface and inside the cell, producing a strong fluorescent signal. In viable cells, the dye reacts only with surface amines, resulting in significantly lower fluorescence intensity. For this analysis, cells negative for the LIVE/DEAD stain were considered viable, based on our gating strategy. The results obtained confirm the good biocompatibility of the system under investigation: under all experimental conditions tested, including the longest exposure times (24 h) and the highest concentrations of nanoparticles (50 μg/mL), the percentage of viable cells remained consistently above 90% (Figure 10).

**Figure 10:**
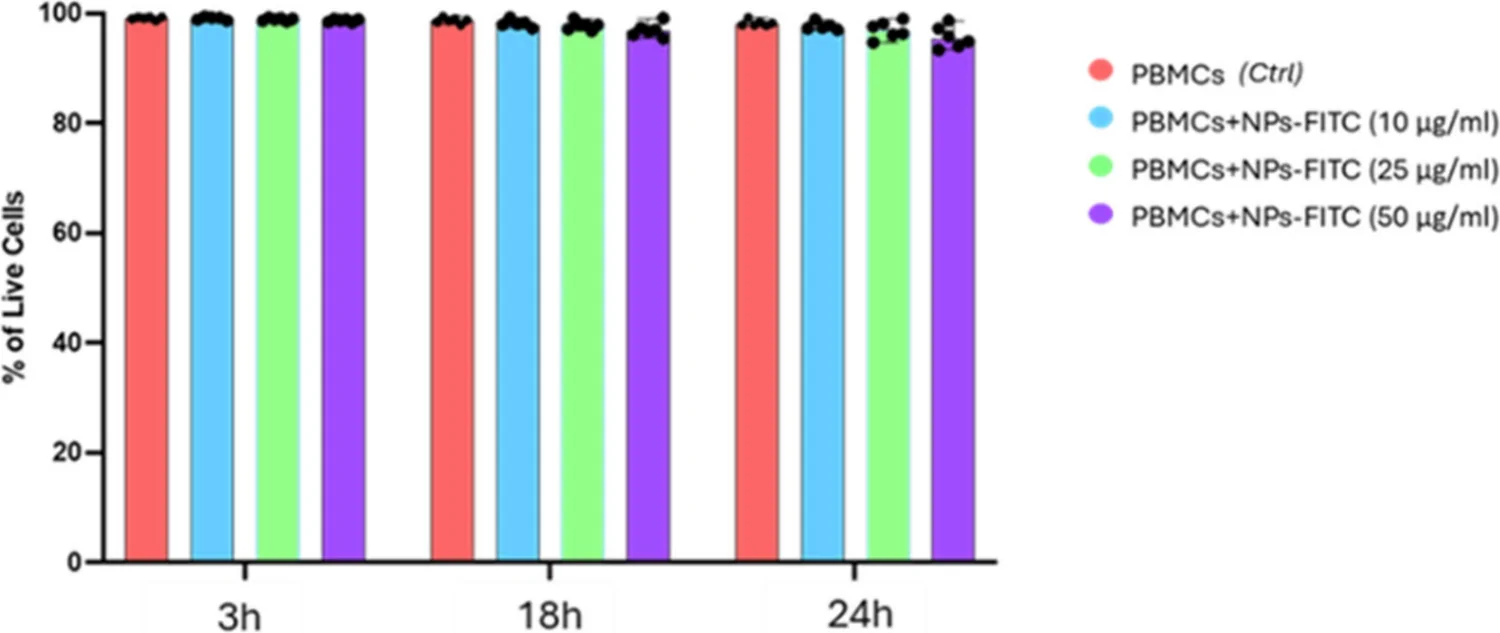
Frequency of live cells following exposure to different concentrations of AuNPs-cit-l-cys-FITC at three time points (3, 18, and 24 h). Cell viability remains stable over time for all tested concentrations: 10 μg/mL (light blue), 25 μg/mL (green), and 50 μg/mL (brown), and is comparable to that of the untreated control cells (red).

Overall, these data suggest that our goal has been achieved and that AuNPs-cit-l-cys-FITC can be chosen as the best drug delivery system compared to others, since they not only possess excellent fluorescent labeling properties but also show a favorable biocompatibility profile in the range of 10–50 μg/mL for up to 24 h.

## 4 Conclusions

In this work, the synthesis of gold and silver nanoparticles functionalized with different biocompatible ligands, such as polyethylene glycol and the citrate-cysteine pair, was optimized to modulate their colloidal stability and surface reactivity. TEM analyses confirmed their nanosize, and DLS and Z-potential measurements demonstrated their hydrodynamic dimensions and colloidal stability, highlighting differences between the systems. The nanoparticles were subsequently functionalized with FITC, a fluorescent dye, to enable their detection and tracking within PBMCs using flow cytometry. The AuNPS-cit-cys-FITC particles were found to be the most suitable, based on their stability, and were also found to be highly biocompatible following cell survival tests (AuNPs-cit-l-cys in the range 10–50 10 μg/mL) with PBMCs. All these results highlight the high potential of this system for applications in drug delivery and bioimaging, particularly for multiple sclerosis treatments.

## Supplementary Material



## Notes

SR-XPS data analysis results (Table S1); FT-IR peaks position and assignments (Table S2); UV–vis spectra of AuNPs-PEG synthesized using different molar ratios of PEG-SH to gold (Figure S1); ⟨2*R*_H_⟩ of Au-CitCys NPs measured in water and in RPMI medium supplemented with 5% human serum at 25 and 37 °C (Figure S2); SR-XPS spectra collected at C 1s core-level for AuNPs-cit-Lcys-FITC, AuNPs-PEG -FITC, AgNPs-cit-Lcys-FITC, AgNPs-PEG-FITC (Figure S3); C 1s, S 2p, O 1s core-level SR-XPS spectra collected on PEG-SH (Figure S4); SR-XPS spectra collected at Au 4f or Ag 3d core-level for AuNPs-cit-Lcys-FITC, AuNPs-PEG -FITC, AgNPs-cit-Lcys-FITC, AgNPs-PEG-FITC (Figure S5); SR-XPS spectra collected at O 1s core-level for AuNPs-cit-Lcys-FITC, AuNPs-PEG -FITC, AgNPs-cit-Lcys-FITC, AgNPs-PEG-FITC (Figure S6) (PDF)

## Abbreviations

CCR2: CC chemokine receptor 2
CCL2: CC chemokine ligand 2
CCR5: CC chemokine receptor 5
TLC: thin layer chromatography
