# Supplementary material for: Fluorescent Biofunctionalized Silver and Gold Nanoparticles: Bright Tools for Bioimaging Applications
Source: ACS Omega. 2026 Sep 13;11(38):57830–44. doi: 10.1021/acsomega.6c07048 (PMC13628621; doi:10.1021/acsomega.6c07048)
Supplement: Supplementary file 1 [file ao6c07048_si_001.pdf]

Supporting Information

Fluorescent biofunctionalized silver and gold nanoparticles: bright tools for bioimaging applications

*Elena Olivieri[1,2], Federica Bertelà[1,3], Sofia Lemaire[1], Miranda Parisi[1], Luca Battistini[2], Giovanna Borsellino[2], Daniela F. Angelini[2],Edyta Beyer[4], Barbara Wolanin[4], Tomasz Sobol[4], Valentin-Adrian Maraloiu[3], Giovanna Iucci[1], Chiara Battocchio[*1], Iole Venditti[1]*

[1]Roma Tre University, Department of Sciences, via della Vasca Navale 79, 00146, Rome Italy

[2]Neuroimmunology Unit, Santa Lucia Foundation IRCCS, Rome 00143 (IT)

[3]National Institute of Materials Physics, 405A Atomistilor St., 077125 Bucharest, Romania

[4]National Synchrotron Radiation Centre SOLARIS, Jagiellonian University, 30-392 Kraków, Poland

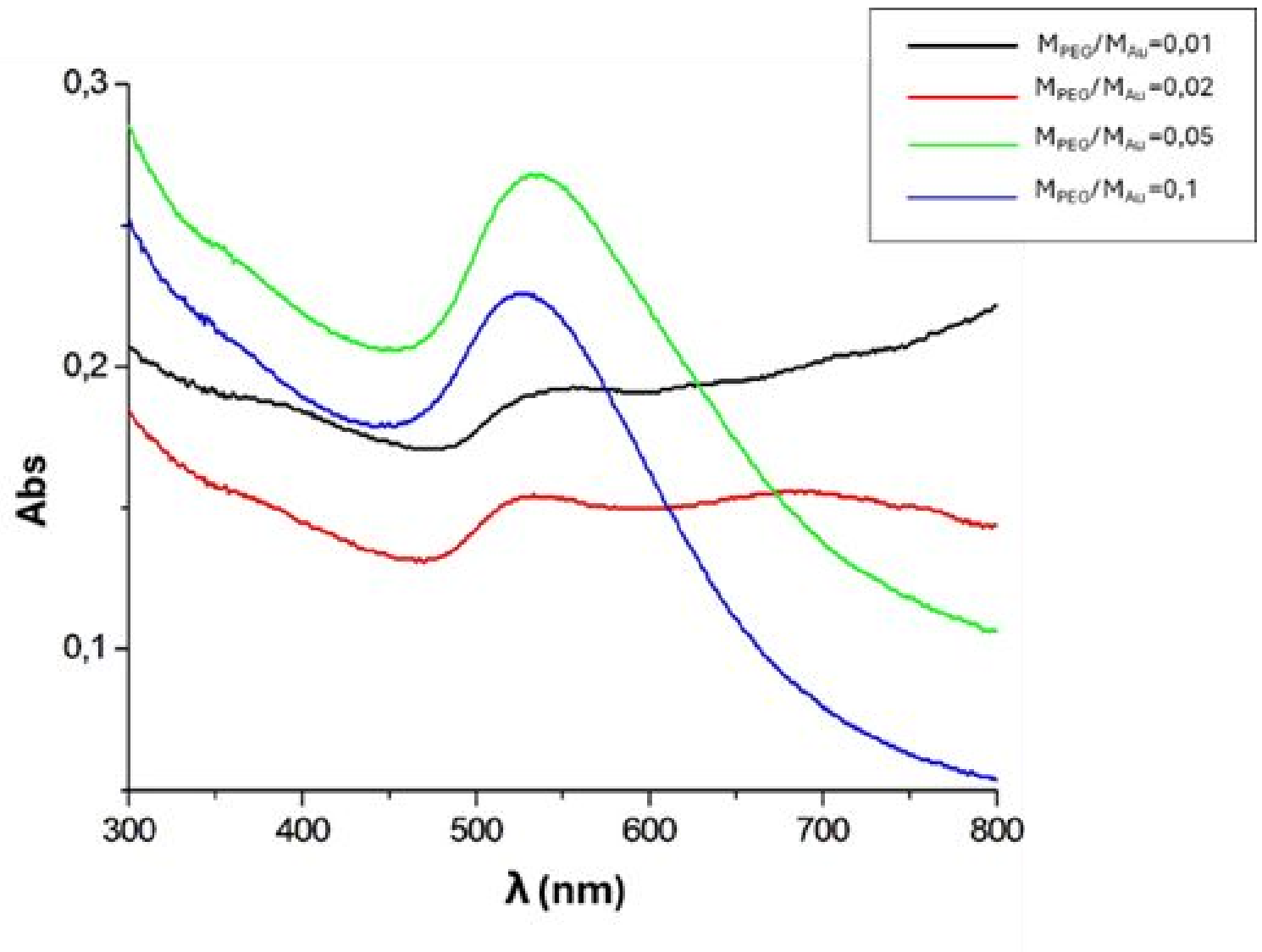

Figure S1 UV-Vis absorption of AuNPs-PEG in water solution, synthesized with varying molar ratios of PEG to gold precursor (M_{PEG}/M_{Au}). In black $M_{PEG}/M_{Au=}$ 0.01; in red $M_{PEG}/M_{Au=}$ 0.02; in green $M_{PEG}/M_{Au=}$ 0.05; in blue $M_{PEG}/M_{Au=}$ 0.1. Black and red curves are NPs with low PEG concentrations. Green and blue curves are NPs were the ratio is highest.

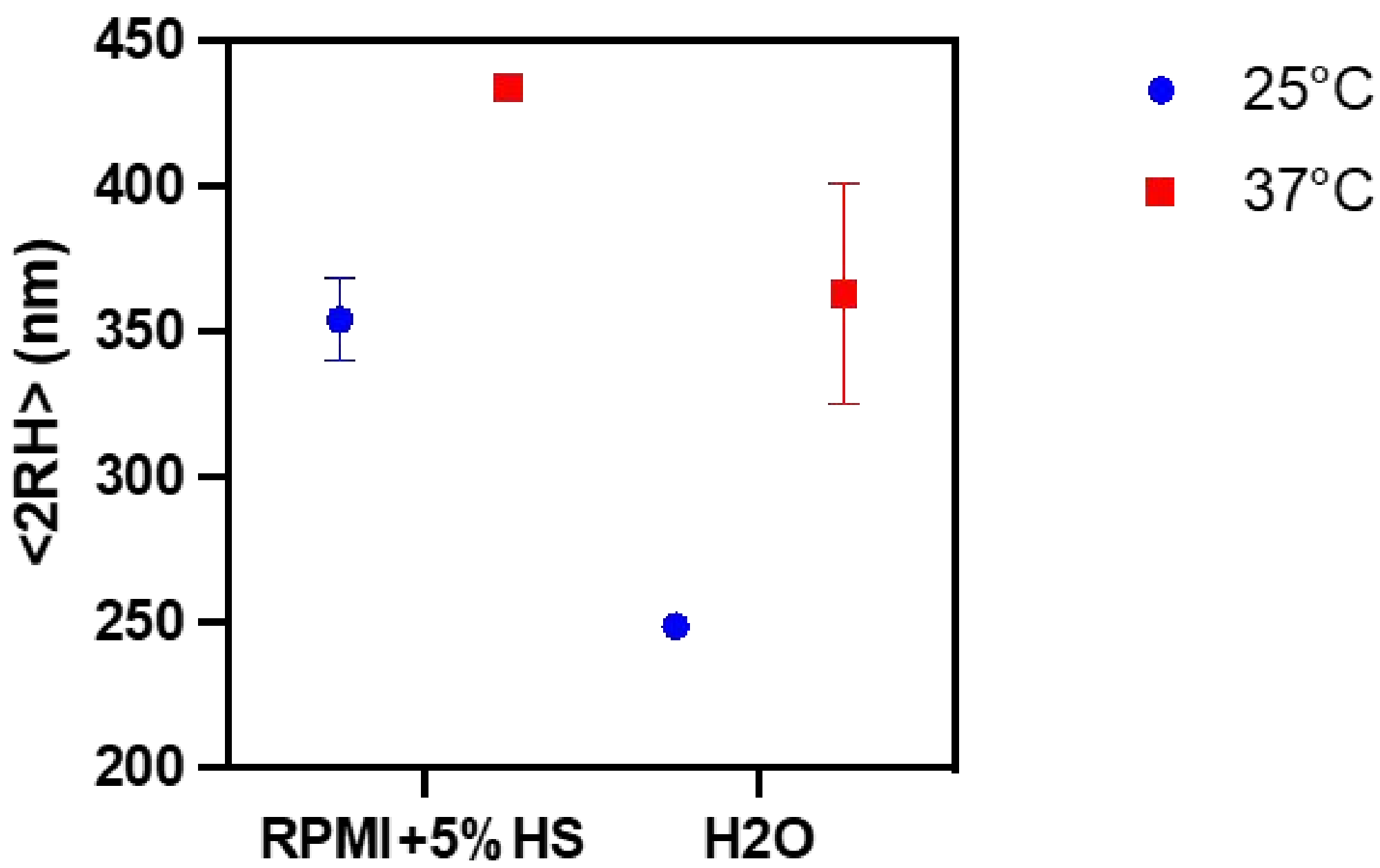

Figure S2 <2R$_H$> of Au-CitCys NPs measured in water and in RPMI medium supplemented with 5% human serum (HS) at 25 °C (blue) and 37 °C (red). Each point represents the average of at least 3 values and the standard deviation, represented as an error bar which is sometimes not appreciable.

SAMPLE	SIGNAL	BE (eV)	FWHM (eV)	Atomic % (exp)	ASSIGNMENT
AuNPs-Lcys-cit	C1s	285,0	1,5	90%	C-C
		286,7		5%	C-N, C-S
		288,4		3%	C-O
		290,0		2%	COOH
	S2p $3_{/2}$	161,3	1,0	45%	Au-S (sp)
		162,1		28%	Au-S (sp^3)
		163,3		17%	S-S
		164,0		10%	RS-H
	Au4f $_{7/2}$	83,8	0,6	92%	Au(0)
		84,4		8%	Au(δ+)
	N1s	397,9	1,5	38%	NH_2 - TiO_2
		399,1		46%	NH_2
		400,3		16%	NH_3^+
	O1s	530,6	1,3		Ti-O_2
		531,7		74%	C=O
		532,5		26%	C-O
AuNPs-Lcys-cit-FITC	C1s	285,0	1,5	88%	C-C
		286,6		7%	C-N, C-S
		288,2		3%	C-O
		289,7		2%	COOH
	S2p $_{3/2}$	161,3	1,0	49%	Au-S (sp)
		162,3		29%	Au-S (sp^3)
		164,0		22%	RSH, S-S
		167,5			oxidized S
		169,6			oxidized S
	Au4f $_{7/2}$	83,8	0,5	91%	Au(0)
		84,4		9%	Au(δ+)
	N1s	398,0	1,5	18%	NCS
		399,1		60%	RNH_2
		400,4		22%	NH_3^+
	O1s	529,9	1,3		TiO_2
		531,0		74%	C=O
		531,8		26	C-O
AuNPs-PEG	C1s	285,0	1,3	87%	C-C
		286,5		7%	C-N, C-S
		288,1		3%	C-O
		289,6		3%	COOH
	S2p $_{3/2}$	160,7	1,1	28%	Au-S (sp)
		162,0		33%	Au-S (sp^3)
		162,7		26%	S-S
		164,6		13%	RS-H
		167,7			oxidized S
		169,9			oxidized S
	Au4f $_{7/2}$	83,8	0.6	86%	Au(0)

		84.4		14%	Au(δ+)
	N1s	398,8	1,7	70,5%	RNH_2
		400,3		29,5%	RNH_3^+
	O1s	531,4	1,5	18%	C=O
		532,8		82%	C-O
		534,0			H_2O
AuNPs-PEG-FITC	C1s	285,0	1,3	91%	C-C
		286,7		5%	C-N, C-S
		288,0		2%	C-O
		289,4		2%	COOH
	S2p $_{3/2}$	160,7	1,2	22%	Au-S (sp)
		162,1		51%	Au-S (sp^3)
		163,2		6%	S-S
		164,3		20%	RS-H
		167,4			ox S
		169,5			ox S
	Au4f $_{7/2}$	83,8		92%	Au(0)
		84,4		8%	Au(δ+)
	N1s	398,0	1,5	36%	NCS
		399,0		47%	RNH_2
		400,3		17%	NH_3^+
	O1s	529,8	1,5		TiO_2
		531,2		87%	C=O
		532,5		13%	C-O

SAMPLE	SIGNAL	BE (eV)	FWHM (eV)	Atomic % (exp)	ASSIGNMENT
AgNPs-Lcys-cit	C1s	285,0	1,3	93%	C-C, C-S, C-N
		286,3		6%	C-O
		287,7		2%	COOH
	S2p	160,6	1,0	32%	Ag-S (sp)
		161,5		42%	Ag-S(sp^3)
		162,9		21%	S-S
		164,4		5%	RS-H
	Ag3d $_{5/2}$	367,8	0,7	95%	Ag(0)
		368,7		5%	Ag(δ+)
	N1s	399,0	2,5	50%	RNH_2
		400,7		50%	RNH_3^+
	O1s	531,0	2,0	21%	C=O
		532,2		79%	C-O
AgNPs-Lcys-cit-FITC	C1s	285,0	1,2	91%	C-C
		286,4		4,5%	C-N; C-S
		288,3		2%	C-O
		289,6		2,5%	COOH
	S2p $_{3/2}$	160,3	1,1	16%	Ag-S(sp)
		161,5		20%	Ag-S(sp^3)
		164,2		4%	RS-H
		167,1			oxidised S
		169,8			oxidised S

		170,8			oxidised S
	Ag3d	367,6	0,5	83%	Ag(0)
		368,1		17%	Ag(δ+)
	N1s	397,9	1,7	8%	RNCS
		399,0		64%	RNH_2
		400,2		28%	RNH_3^+
	O1s	531,2	1,9	33%	C=O
		532,8		67%	C-O
AgNPs-PEG	C1s	285,0	1.3	90%	C-C
		286,4		4%	C-N, C-S
		388,1		2%	C-O
		289,7		4%	COOH
	S2p $_{3/2}$	160,2	0,8	58%	Ag-S (sp)
		161,4		24%	Ag-S (sp^3)
		162,6		12%	RS-SR
		163,4		6%	RS-H
		167,1			oxidised S
		169,8			oxidised S
		170,8			oxidised S
		171,8			oxidised S
	Ag3d $_{5/2}$	367,4	0,6	91%	Ag(0)
		368,2		9%	Ag(δ+)
	N1s	397,9	1,5	8%	NH_2-Ti
		398,9		70%	RNH_2
		400,3		22%	RNH_3^+
	O1s	529,8	1,5		TiO_2
		531,3		70%	C=O
		532,8		30%	C-O
AgNPs-PEG-FITC	C1s	285,0	1,4	91%	C-C
		286,4		4%	C-N, C-S
		288,4		2%	C-O
		289,8		3%	COOH
	S2p $_{3/2}$	160,4	1,2	49%	Ag-S (sp)
		161,9		37%	Ag-S (sp^3)
		163,8		14%	RS-H
		167,2			oxidised S
		170,7			oxidised S
		171,9			oxidised S
	Ag3d $_{5/2}$	367,8	0.7	79.5%	Ag(0)
		368,6		20.5%	Ag(δ+)
	N1s	398,2	1,5	14%	NCS
		399,2		72%	RNH_2
		401,0		13%	NH_3^+
	O1s	530.56	1.50		TiO_2
		532.82		85%	C=O
		533.04		15%	C-O
PEG-SH	C1s	285,0	1,1	68%	C-C

		285,9		22%	C-S
		286,9		8%	C-O
		288,1		2%	COOH
	$S2p_{3/2}$	160,6	1,4	13%	Au-S (sp)
		161,8		18%	Au-S (sp^3)
		163,2		20%	RS-SR
		165,6		50%	RS-H
	O1s	531,3	1,6	16%	C-O-C
		532,8		84%	C-OH
		534,1			H_2O

Table S1. Complete collection of XPS data: BE (eV), FWHM (eV), atomic percentage values and proposed assignments

assignment	peak position (cm^{-1})		
	FITC	AgNPs-cys-FITC	AgNPs-PEG-FITC
O-H stretching	3377	3400	3600
N-H stretching		3240	3200
C-H stretching	3100-2900	2980	2980
N=C=S stretching	2050		
FITC C=O stretching	1754	1751	1750
C=O, C=S stretching		1654	1685
aromatic C=C stretching	1635	1623	1635, 1610
N-H bending		1577	
aromatic C=C stretching	1520,1480,1400	1516, 1475, 1400	1520 (w), 1420
phenol O-H bending	1230	1275	1290
ester C-O stretching	1170	1155	1135
ether C-O stretching	1040	970	1020
aromatic C-H bending	870, 787, 730	855, 780, 735	840 (sh) 745

Table S2. FT-IR peaks position and assignments

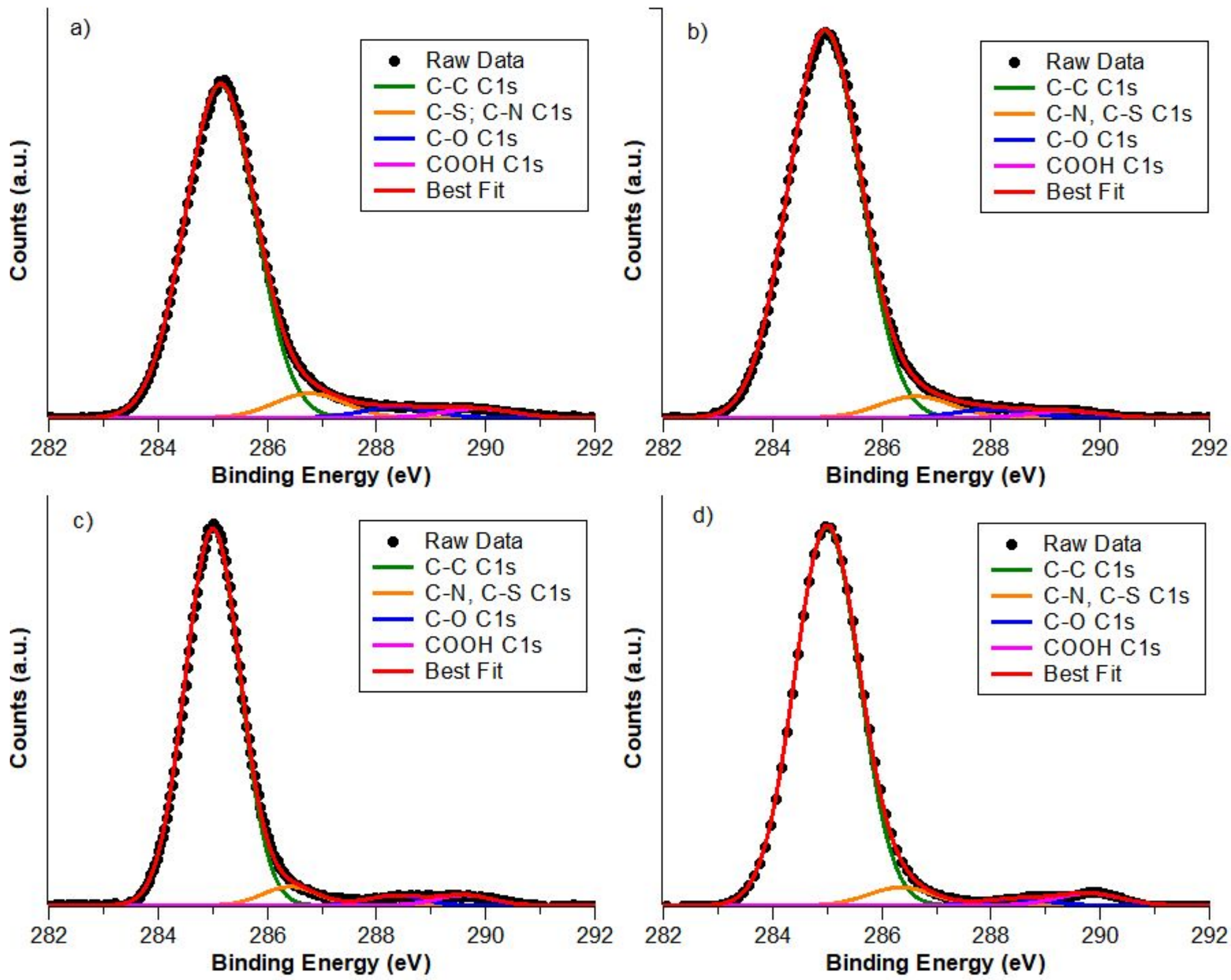

Figure S3. Above: C1s core-level spectra collected on a) AuNPs-cit-Lcys-FITC, b) AuNPs-PEG -FITC; c) AgNPs-cit-Lcys-FITC, d) AgNPs-PEG-FITC.

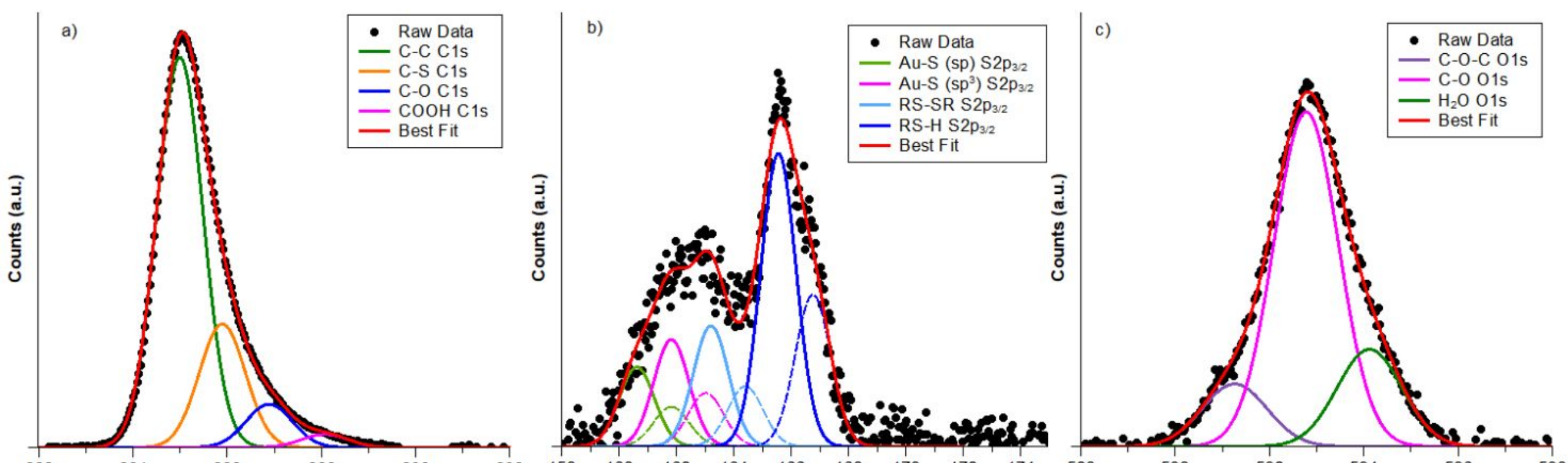

Figure S4. A) C1s; b) S2p; c) O1s core-level spectra collected on PEG-SH

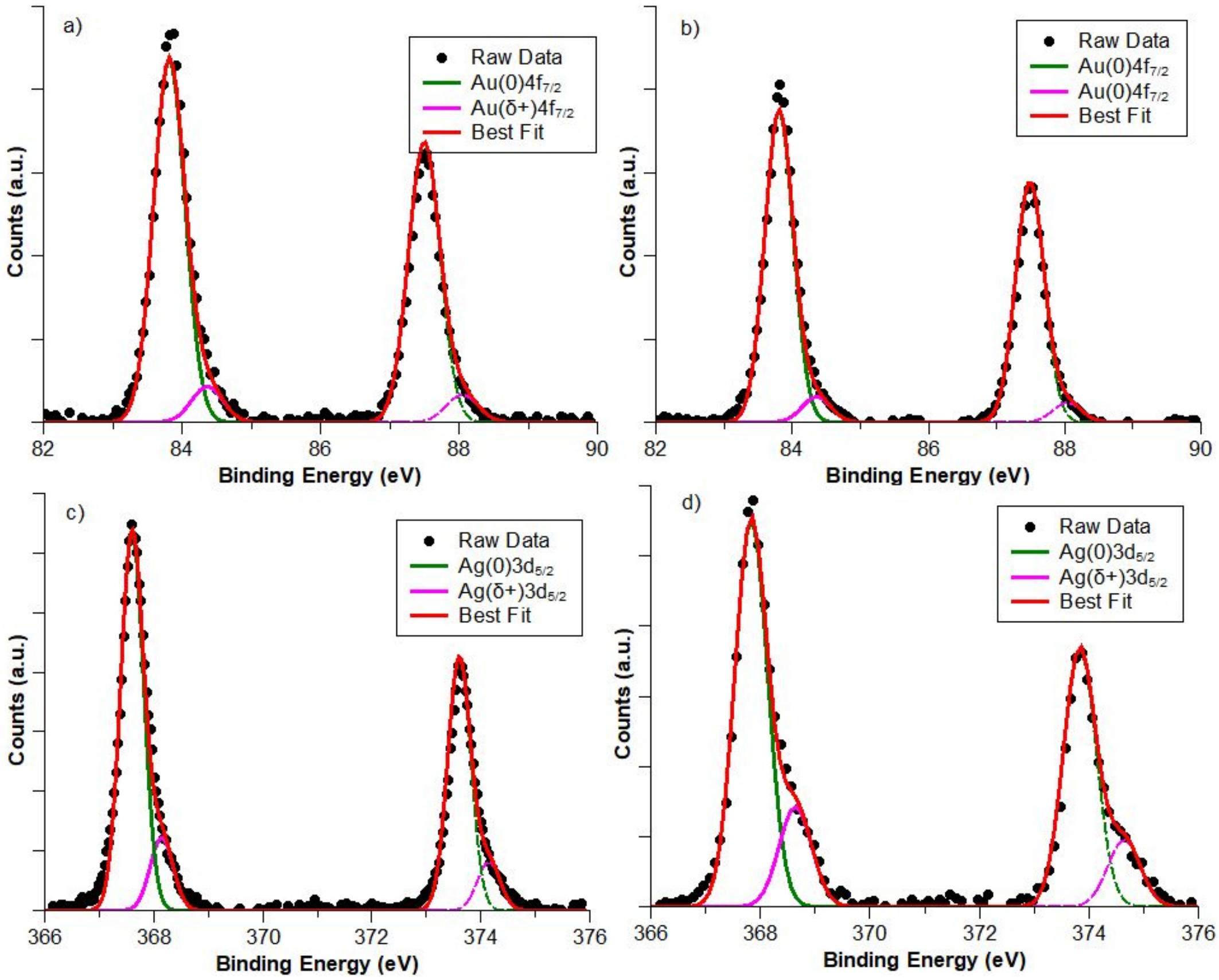

Figure S5. Above: Au4f core-level spectra collected on a) AuNPs-cit-Lcys-FITC, b) AuNPs-PEG -FITC; below: Ag3d core-level spectra collected on c) AgNPs-cit-Lcys-FITC, d) AgNPs-PEG-FITC.

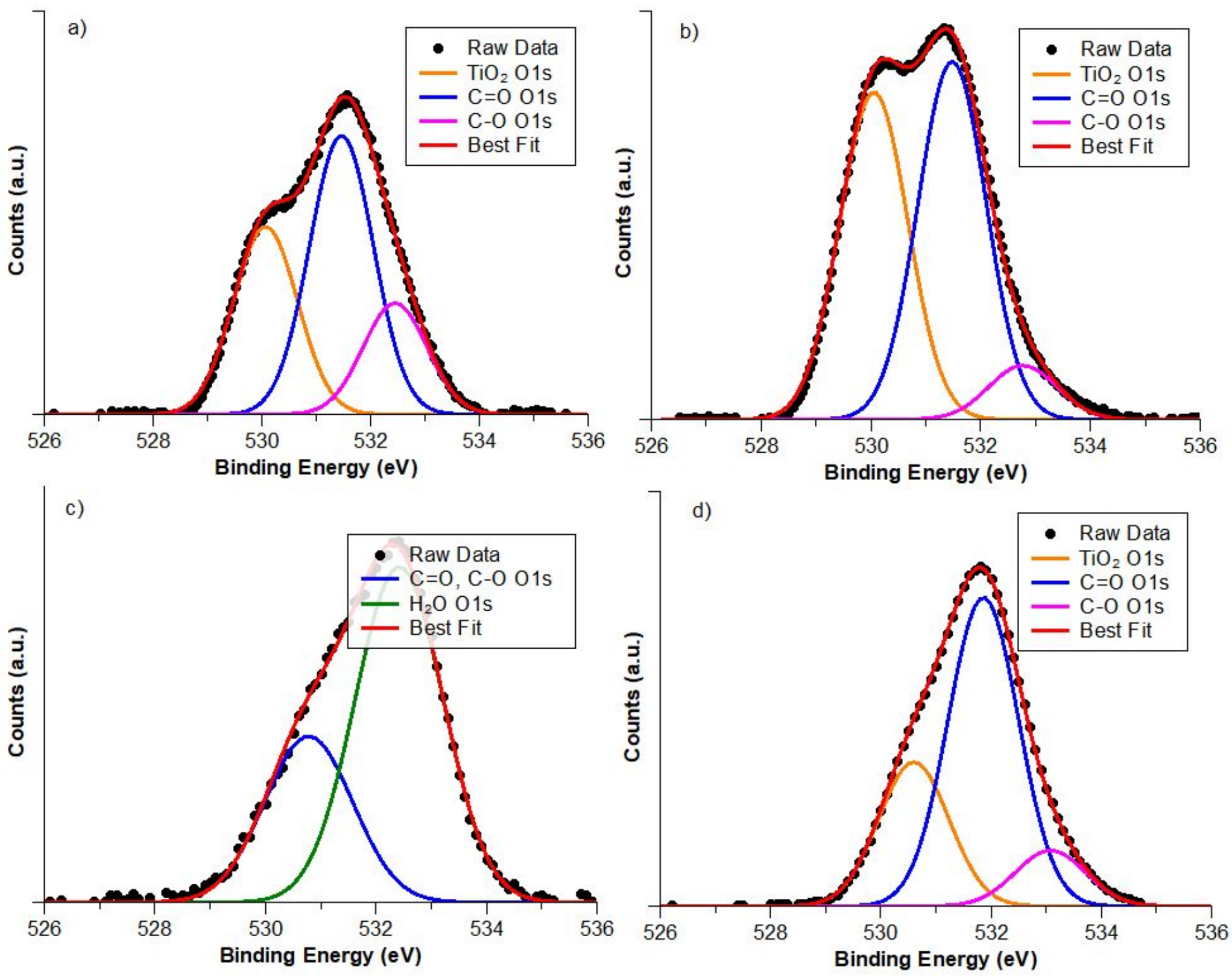

Figure S6. Above: O1s core-level spectra collected on a) AuNPs-cit-Lcys-FITC, b) AuNPs-PEG -FITC; c) AgNPs-cit-Lcys-FITC, d) AgNPs-PEG-FITC.
